# A green approach to antibacterial and antioxidant wool and polyamide 6 fabrics through bioactive *Aspergillus turcosus* extracted pigment for healthy and high-performance textile products

**DOI:** 10.1038/s41598-026-55888-w

**Published:** 2026-06-19

**Authors:** Marwa Abou-Taleb, Rania A. Zaki, Sanaa K. Gomaa

**Affiliations:** 1 Proteinic and Man‑made Fibres Department, Textile Research and Technology Institute, National Research Centre, P.O.12622, Dokki, Giza, Egypt; 2 Chemistry of Natural and Microbial Products Department, Pharmaceutical and Drug Industries Research Institute, National Research Centre, P.O.12622, Dokki, Giza, Egypt

**Keywords:** *Aspergillus turcosus*, Antibacterial, Antioxidant, Wool, Polyamide 6, Fabric, Biotechnology, Chemistry, Materials science

## Abstract

Wool is a proteinic fiber with unique properties like breathability, excellent moisture management, and odor resistance; these features make it highly desirable for next-to-skin applications. Polyamide 6 (PA6) fabric as well is a strong, durable, and versatile fabric. Recently natural dyes from plants and microorganisms with predominant properties have been widely used in textile coloration to avoid synthetic dyes, which are often petroleum-based. This study is the first investigation into the pigment-producing capability of *Aspergillus turcosus*, a strain that hasn’t been associated with this activity before. The extracted pigment has been used in dyeing wool and polyamide 6 fabrics at different reaction conditions. The chemical characterization for the functional groups of the extracted pigment has been identified using Fourier Transform Infrared Spectroscopy *(FTIR)* and Nuclear Magnetic Resonance analysis *(NMR)*. The colorimetric data were assigned for the dyed fabrics, and the results showed that the extracted pigment showed a good affinity to the fabrics with good colorfastness properties. Antibacterial activity was assigned, and the data revealed an increase in the inhibition zone diameter from 0 to 31 mm for wool fabric and from 0 to 35 mm for PA6 fabrics. Additionally, the dyed fabrics showed enhancement in the antioxidant inhibition rate from 15 to 60% for wool fabric and 10 to 45% for PA6 fabrics compared to the undyed ones. The extracted pigment also enhanced the UPF of the dyed fabrics by 65% and 85% for wool and PA6, respectively.

**Supplementary Information:**

The online version contains supplementary material available at 10.1038/s41598-026-55888-w.

## Introduction

Wool fabric is well-known for its customary roles in apparel and home textiles due to its versatile properties, such as resisting dirt, and its ability to absorb moisture prevents the accumulation of static electricity, and therefore wool does not attract lint and dust from the air. It is also increasingly recognized for its unique inherent properties and flexibility that make it a suitable candidate for a growing range of medical applications, from wound dressing to therapeutic textiles^1^.

Another fabric of interest is polyamide fabric; it is a well-known synthetic fabric and recognized for its strength, elasticity, and adaptability. These properties increase its applications in the medical sector, from sutures and surgical meshes to compressed garments and advanced wound dressings^2^.

Despite their beneficial properties for definite medical applications, both wool and polyamide 6fabrics in their native states have several disadvantages that hinder their extensive use in the medical sector. For example, wool has a potential for allergenicity and irritation due to lanolin and coarse fiber^3^, its tendency to felt and shrink, and limited sterilizing methods due to protein structure^4,5^. In addition to that, both wool and polyamide exhibit poor inherent antibacterial properties and limited antioxidant activity, which necessitate specific treatments to enhance their functionality for medical applications^6,7^. Wool and polyamide 6 differ considerably in their chemical structure and the accessibility to the functional group, which governs their interaction with different materials, where wool keratin contains ionizable amino acid residues, while PA6 exhibits higher crystallinity and limited reactive sites.

There were many tries to avoid the inherent disadvantages accompanied by wool and polyamide 6 fibers. For example, wool fibers were successfully treated for felt-proofing using an ecologically acceptable and energy-saving plasma technique followed by treatment with biopolymer^8^, or by using tailored immobilized proteolytic thermozymes^9^. A lot of antimicrobial agents, such as metals and metal compounds, metal nanoparticles, quaternary ammonium salts, poly(hexamethylenebiguanide), triclosan, chitosan, dyes, regenerable N-halamine compounds, peroxyacids, and amino coumarins, have been utilized in the textile industry to increase the applications of wool and polyamide 6 fabrics in the medical sector by enhancing properties like conductivity and antimicrobial properties^10–12^. Also, flavonoids extracted from natural sources viz., baicalin, quercetin, and rutin were utilized to enhance the antioxidant and antibacterial properties of polyamide fibers via adsorption technology^13^.

Textile dyes can be divided into two main categories: natural and synthetic dyes. Wool and polyamide fabrics can be dyed with several classes of dyes, and each class offers different properties and different applications. Wool fabrics have an affinity to acid, basic, reactive dyes (as synthetic dyes) and different pigments in addition to their affinity to natural dyes^14,15^. Polyamide fabric as well exhibits a good affinity for acid dyes; disperse dyes, and basic dyes as well as natural dyes^16^.

Conventional dyeing processes usually require the use of a large amount of water and a lot of hazardous chemicals, and consequently generate a considerable amount of wastewater, causing significant environmental health and safety concerns.

Recently, researchers have studied the less water or waterless dyeing technologies in textile dyeing. For example, in the linear silicon (LS) dyeing system, the dye uptake showed more than 99% and above 80% fixation rate, and small solid waste emission^17^. Additionally, the low-pressure waterless dyeing processes, a low molecular weight by-product i.e., polyester, oligomers formed by polyester condensation reaction result in a significant color difference, as the oligomers existing inside and on surfaces affect the quality of dyed products^18^.

The salt-free and less-water reactive dyeing technology promotes green and low-carbon development of textile dyeing industry, fulfilling human development needs while minimizing pollution.

Natural dyes, also known as natural pigments, are mainly derived from plants, fruits, animals, microorganisms, or naturally colored ores, while synthetic dyes are extracted from petroleum and coal tar. Owing to the harmful effects of synthetic colorants, there is global interest in developing colorants from natural sources for textile dyeing to avoid the negative impacts of auxiliaries as well as chemicals used in synthetic dyes^19^.

Colorants extracted from the natural resources are thought to be safer than synthetic dyes because they are biodegradable, non-carcinogenic, and non-toxic. Moreover, natural dyes are more accepted due to their softer color shades, deodorizing and anti-cancer properties. Besides many of them have antioxidant properties and are considered antibacterial and anti-inflammatory compounds^20^.

Because of their stability and the availability of developing technologies, microbial pigments are quite interesting^21^. In recent years, pigments from fungal genera such as *Aspergillus*, *Penicillium*, *Monascus*, and *Talaromyces*, in particular, have become environmentally friendly alternatives to synthetic colorants in different industries, such as food, textiles, and cosmetics. The fungal pigments have several benefits, including simplicity of manufacture, affordability, water solubility and possible health advantages. Fungi are a feasible source for the industrial manufacture of pigments because they can be grown on a big scale at a low cost. A*spergillus turcosus* is a filamentous fungus belonging to the group of *Ascomycota* and the genus *Aspergillus*. It is taxonomically distinguished from closely related species based on colony morphology, microscopic features, and DNA sequence data. They produce a wide range of secondary metabolites, but pigment investigations for textile applications have been limited^22^.

Microorganisms like bacteria and fungi have the ability to produce colorants and are open for bulk production. Bacteria like C*hromobacterium violaceum*,* Serratia marcescens* and *Chryseobacterium* sp. are capable of producing violet, red and yellow-orange pigments^23^, and *Penicillium* sp. produces ankaflavin, which has a strong affinity for wool^22^.

Literature has also recorded many attempts to dye textiles with natural dyes. For example, wool fabrics were also pad-dyed using color paste containing natural dyes prepared from curcumin dispersion, and the dyed wool fabrics exhibited improved antibacterial activity against *E. coli* and *S. aureus*. T^24^.

Natural silk and wool fabrics were dyed by *Azollapinnata* extracts with a novel cumin color resulting in higher UV protection, enhanced antimicrobial activity, and antioxidant properties in the fabrics^25^.

Polyamide fabrics were also dyed with madder and safflower yellow natural dyes, and the dyed fabrics showed enhancement in the UV-protection factor and antibacterial properties of the fabric^26^.

It was reported that natural fabrics, viz., wool, cotton and silk, upon using natural dyes extracted from plants, viz., peony, clove, pomegranate, and gallnut, have excellent *anti-Staphylococcus aureus* activity^27^. Moreover, some previous studies have demonstrated that definite natural dyes such as madder and safflower yellow are able to improve UV protection and antimicrobial effects in textiles^28^.

Generally, natural dyes have limited affinity for the fibres; that is why using mordant is essential to enforce the fixation of the colorant on the fibre via the formation of the complex with the dye.

A mordant is a chemical substance which can be fixed on the fibre and also forms a chemical bond with the natural dyes. It acts as a bridge between the dye and the fiber, improving the dye’s ability to penetrate and bind to the fabric in addition to enhancing the fastness properties of the dyed fabrics. The most important mordants that have been used are alum sulphate, ferrous sulphate, copper sulphate, zinc sulphate, and tannic acid^29^.

This work aims at using a natural pigment extracted from *Aspergillus turcosus*in dyeing wool and polyamide 6 fabrics and acquiring those fabrics new functional properties viz., enhanced antibacterial properties and antioxidant activity, in addition to good colorfastness properties.

## Materials and methods

### Materials

Plain weaved crossbred wool fabric 280 g/m^2^with density 168 thread/cm, wrap count = 6.46 Ne and weft count = 6.46,and plain weaved structure polyamide 6 (PA6)fabric 180 g/m^2^ with density = 616 thread/cm, wrap count = 17.688 Ne and weft count = 15.76 Ne were supplied by Misr Company for Spinning and Weaving, Egypt.

Aluminum sulphate as a mordant was purchased from ADWIC, El-Nasr pharmaceutical chemicals Co. Buffer solutions of pH 4 (citric acid/sodium hydroxide/sodium chloride solutions), pH 7 (potassium dihydrogen phosphate/disodium hydrogen phosphate), and pH 9 (sodium tetraborate buffer solution) were purchased from Fluka, Germany.Potato dextrose agar and potato dextrose broth media were purchased from Sigma Aldrich, USA.

### Methods

#### Isolation and characterization of pigment producing fungi

The fungal isolates were collected from soil samples obtained from National Research Center, Egypt. One gram of soil samples was suspended in100 ml distilled water, shacked at 37 °C for 15 min, then diluted up to 10^− 6^ and cultured on potato dextrose agar medium containing ceftriaxone (1000 mg) as an antibacterial. The Plates were incubated at 30 °C for 7 days. After incubation time, fungal isolates exhibiting reverse pigmentation were refined and stored on PDA slants in a refrigerator at 4 °C for the subsequent pigment extraction test.

##### Molecular identification

The 16 S rRNA PCR gene amplification was carried out using Maxima^®^ Hot Start PCR Master Mix (Thermo Scientific, K1051), and genomic DNA was extracted using the Wizard^®^ Genomic DNA Purification Kit (Promega, Southampton, UK) according to the manufacturer’s instructions provided by Sigma Company of Scientific Services, Egypt (www.sigma-co-eg.com). The identified sequences were evaluated using the BLAST algorithm and compared against the sequences available in the National Center for Biotechnology Information’s (NCBI) Gen Bank database (www.ncbi.nlm.nih.gov) to determine the closest phylogenetic relatives.

##### Pigment production media

For pigment production by the identified fungal strain, 50 ml of potato dextrose broth media (PDB) was inoculated with 3 ml of spore suspension (1 × 10^6^ spores/ml) of selected fungi. The inoculated media was incubated at 30 °C on a rotary shaker at 120 rpm for seven days. A medium without a fungal inoculation was used as a negative control^30^.

##### Pigment extraction

After incubation time, the culture of identified fungi was filtered through Whatman No. 1 filter paper. The filtrate, the extracellular pigment, was exposed to solvent extraction. The culture filtrate has been mixed with an equal volume of methanol (1:1, v/v) and thoroughly stirred with a magnetic stirrer for 30 min at room temperature. The culture filtrate and solvent were taken in a separating funnel and mixed well. Evaporation was used to separate and concentrate the solvent to obtain yellow pigment. UV-Vis scanning between 200 and 800 nm was used to determine the pigment’s maximum absorption peak, while distilled water served as a blank control^31^.

##### Optimization of the culture conditions for yellow pigment production

The following variables (incubation temperature, pH, and incubation time) were evaluated to improve the production of yellow pigment from *Aspergillus turcosus*. The optical densities of the culture filtrates were measured at 430 nm (the pigment’s maximum absorption peak) to know the optimal conditions for the production of pigment. Various temperatures, including 25 °C, 30 °C, 35 °C, 40 °C and 45 °C were used to establish the optimal temperature. The media were adjusted at different pH (5.0, 6.0, 7.0, 8.0, and 9.0) to determine the optimal one for pigment production. The production media was incubated at different incubation times (5, 7,9,11,13 and 15 days) to determine the optimal time for maximum pigment production^32^.

#### Treatment of the fabric

##### Scouring

Wool and polyamide 6 fabrics were scoured to remove any lubricating oils used during the weaving from their surface, a scouring process was carried out by immersing the fabric in an aqueous solution containing Na_2_CO_3_ (2 g/L) and nonionic detergent (1 g/L) for 15 min at 60 °C. The scoured fabric was then removed and rinsed thoroughly with running water and finally dried at room temperature.

##### Mordanting

To minimize experimental variables and focus on affinity of fabrics to the pigment, aluminum sulphate was selected as mordant, as it is commonly used in natural dyeing systems due to its compatibility with the functional groups on the extracted pigment and its ability to form coordination interactions with both wool and polyamide fabrics.

Wool and polyamide 6 fabrics were pre-mordanted using 6% owf aluminum sulphate in a solution with L.R ratio of 1:20 to investigate the effect of mordant on the fastness properties and antibacterial properties of the dyed fabric with natural colorant extracts. Fabric was introduced into the mordant solution at 30℃; for 10 min and then raised to 90℃. Mordanting was continued for 60 min at this temperature. The mordanted wool and polyamide fabrics were washed with running tap water and then dried at room temperature^33^.

##### Wool and polyamide 6 fabric dyeing

**Fig. 1:**
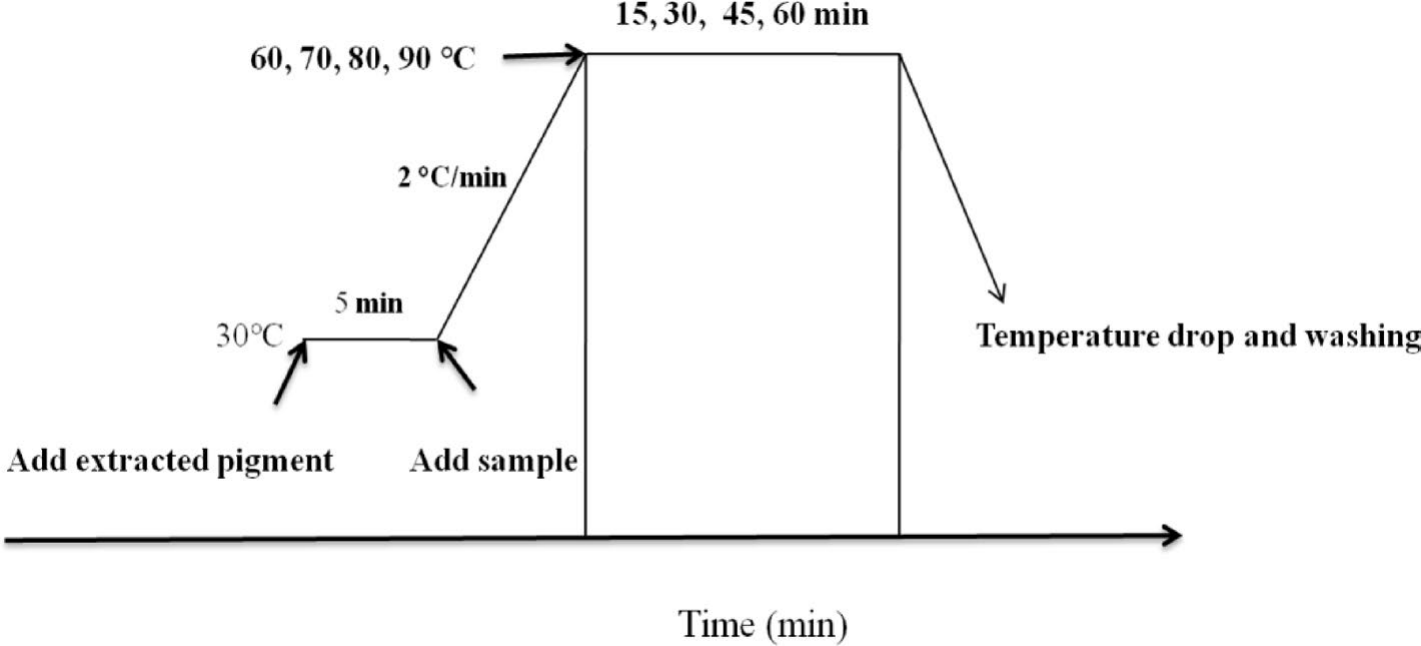
Schematic diagram for the dyeing mechanism for wool and PA6 fabrics.

The affinity of wool and polyamide 6 fabrics to the extracted natural dye was studied using different concentrations (1– 2–3%) shades (i.e. owf) with different pH values (4–7–9), at different temperatures (60–70–80–90 °C) for different periods of time (15–30–45–60 min) min and a liquor ratio 1:50 according to the dyeing diagram (cf. Figure 1)^34^, and the dyed samples were then rinsed with running water, followed by air-drying at room temperature.

### Analyses

#### Color measurements

The color intensity (K/S) values of the dyed wool and polyamide fabrics with the extracted natural pigment were evaluated using a spectrophotometer with pulsed xenon lamps as a light source (Ultra Scan Pro, Hunter Lab, USA) 10° observer with D65 illuminant, d/2 viewing geometry, and measurement area of 2 mm at λ_max_ 450 according to K/S spectra. A modified relative unlevelness index (RUI) at λ_max_ 450 derived from spectroscopic levelness evaluation method reported by Chong et al.^35^, was used to assess the dyeing uniformity.

#### Colorfastness to washing and rubbing

The colorfastness to washing was determined according to method ISO 105-C06 (2010) using Lunder-Ometer^36^. The samples (5 × 10 cm) were sewn between two similar pieces of bleached cotton fabric. The specimen was immersed into an aqueous solution containing 5 g/l non-ionic detergent and 2 g/l of sodium carbonate at a liquor ratio 1:50, the bath was thermostatically adjusted to 45 °C. The test was run for 30 min at 42 r.p.m. the samples were then removed, rinsed twice with occasional stirring or hand squeezing, and then dried. The washing fastness was assessed using the Grey Scale reference for color change. The colorfastness against crocking (both dry and wet) was estimated using the AATCC standard Test Method 8-2016.

#### Colorfastness to light

Colorfastness to light was determined according to AATCC test method (16 A – 1989). The evaluation was established using the blue scale as reference of color change^37,38^.

#### Halochromic test

In the halochromic test, the dyed fabrics were dipped for 30 min in solutions with a pH ranging from 4 to 10. Buffered solutions from Fluka provided the pH values of 4, 7, and 9. The CIELab colorimetric coordinates of the fabrics were subsequently evaluated, and the reference samples used to evaluate the Δ*E* of the fabrics were freshly dyed wool and polyamide 6 fabrics^39^.

#### Nuclear magnetic resonance (NMR) analysis

The proton Nuclear Magnetic Resonance Spectroscopy (^1^H NMR) of the pigment was recorded after suspending the pigment in high-purity deuteron chloroform (CDCl_3_)^40^. ^1^HNMR spectra were obtained in model Bruker High Performance Digital FT-NMR spectrometer Avance III 400 MHz at 20–25 °C, 4.0894465s acquisition time, and 8012.820 Hz spectral width.

#### Fourier transform infrared spectroscopy (FTIR)

The FTIR spectra of the dry extracted pigment, blank, and dyed fabrics were examined by an FTIR spectrophotometer in the region of 4000 –400 cm^− 1^.

#### UV-Vis spectroscopy

UV–Vis spectroscopy was used to examine the dyes absorption pattern in the UV–Visible region using JENWAY-6405 UV/V spectrophotometer (Bibby Scientific Ltd., UK) in the range 300 to 800 nm.

#### X-ray diffraction pattern

The X-ray diffraction pattern was assessed for the blank and dyed fabrics on a Bruker D8 Advance using Cu Ka as the target with a secondary monochromator to operate at 40 KV and 40 mA. The scans were performed within the range of 4 < 2θ < 60 with scanning step of 0.02 in reflection geometry. The crystallinity index (CI) was calculated using the following empirical Eq. (1)^41^:

1 $$\text {CI }\%=\frac {A_{cr}}{A_{total}} \times 100$$

where CI is the crystallinity index, Acr is the area under crystal lattice diffraction, and Aam is the area under the amorphous peaks. In general, a higher CI value indicates higher crystallinity of the sample.

#### Ultraviolet protection factor

The ultraviolet protection factor (UPF) was automatically calculated according to Australia/New Zealand standard AS/NZS-4399:1996 method employing the UPF calculation system of the UV/Vis spectrophotometer as reported in the standard AATCC Test Method 183:2010-UVA Transmittance.

#### Antibacterial activity

The antibacterial activity of pigment and dyed samples, as well as untreated samples, was determined against two bacterial strains, gram-negative bacteria (*Escherichia coli* NRRL-B210) and gram-positive bacteria (*Bacillus subtilis* NRRL-B543,) by the well diffusion method on an agar plate. The test organisms were incubated in the nutrient broth medium at 35 °C for 24 h. Then, 100 µL of the fresh culture of each test organism was separately transferred to nutrient agar plates and carefully spread. Afterward, 50 µL of 100 mg of the pigment extracts/mL DMSO was added to the well. Dyed fabrics and the pristine ones were plated onto the inoculated nutrient agar plate using sterile forceps, and then sterilized glass rings were placed above the samples to ensure contact with the agar. The plates were incubated for 24 h at 35 °C. Antimicrobial activities were evaluated by measuring the inhibition zone diameter (mm).

#### Antioxidant activity

The antioxidant activity of the pigment and dyed samples, as well as untreated, was evaluated using the DPPH free radical scavenging assay as in^42^, with slight modification. Briefly, a 100 µl sample (concentration 50, 100, 150, 200, and 600 µg pigment/ml deionized water) and 2.5 cm² of dyed samples (wool and polyamide) were mixed with 900 µl of 0.1 mM DPPH solution in methanol and incubated in the dark for 30 min at 37 °C. After incubation time, DPPH decolorization was determined by measuring the absorbance at λ_max_ 517 nm. DPPH radical scavenging activity was calculated using the given Eq. 2.

2 $$\text {Inhibition } (\%) = \frac {A_1-A_2}{A1} \times 100$$

where A_1_ was the absorbance of the DPPH solution without the sample and A_2_ was the absorbance of DPPH with the sample.

## Results and discussion

### Isolation and screening of yellow pigmented fungi

**Fig. 2:**
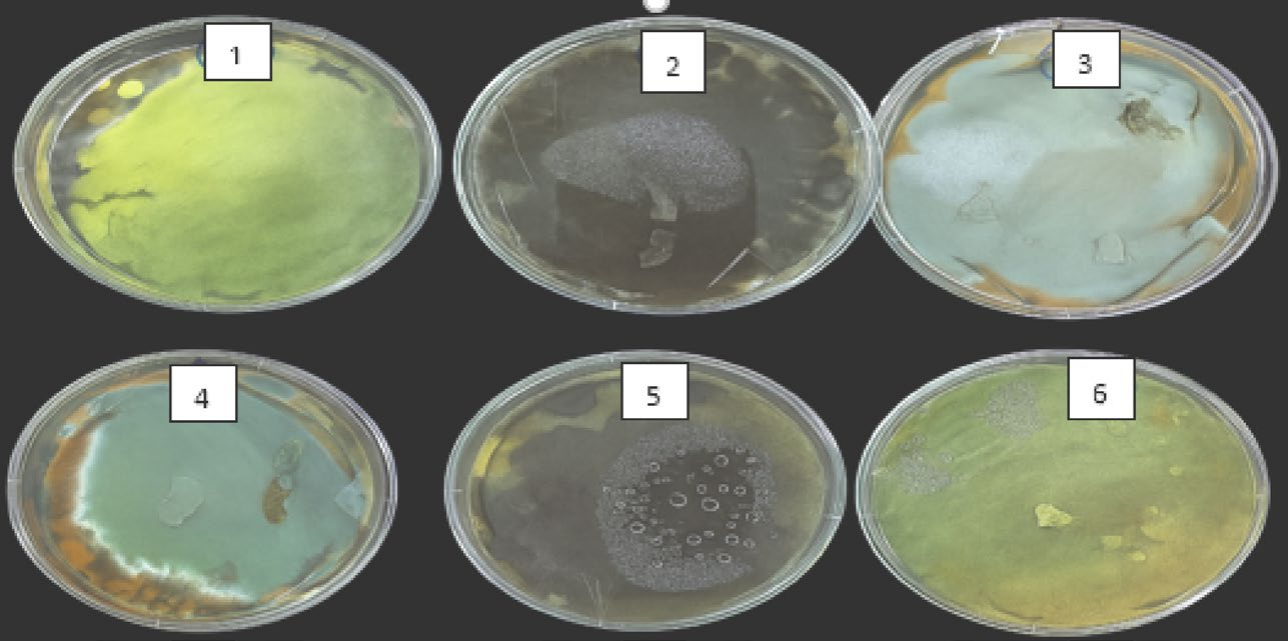
The morphological features of the isolated fungi.

Figure 2 shows six fungal isolates that were screened and grown on PDA, which can be morphologically distinguished by colony color, texture, margin, and growth density. Strong extracellular yellow pigment production potential was suggested by isolate 6. Isolate 6 was chosen for additional research based on its antimicrobial and dye efficiency and its visual pigment output.

### Identification of isolated fungal strain

**Fig. 3:**
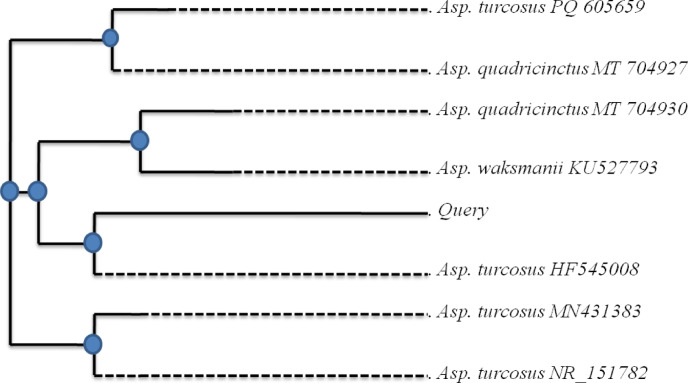
Phylogenetic tree of partial 16 S rRNA sequences of *AspergillusturcosusPX588553*.

According to the 16 S rRNA sequence, the fungal isolate 6 showed the closest resemblance of 100% with the fungal strain *Aspergillus turcosus* (cf. Figure 3).*Aspergillus turcosus* was registered in the Gen Bank with accession number PX588553. To the best of our knowledge, this is the first study on *Aspergillus turcosus* for the production of yellow pigment.

### Production and extraction of yellow pigment from aspergillus turcosus PX588553

**Fig. 4:**
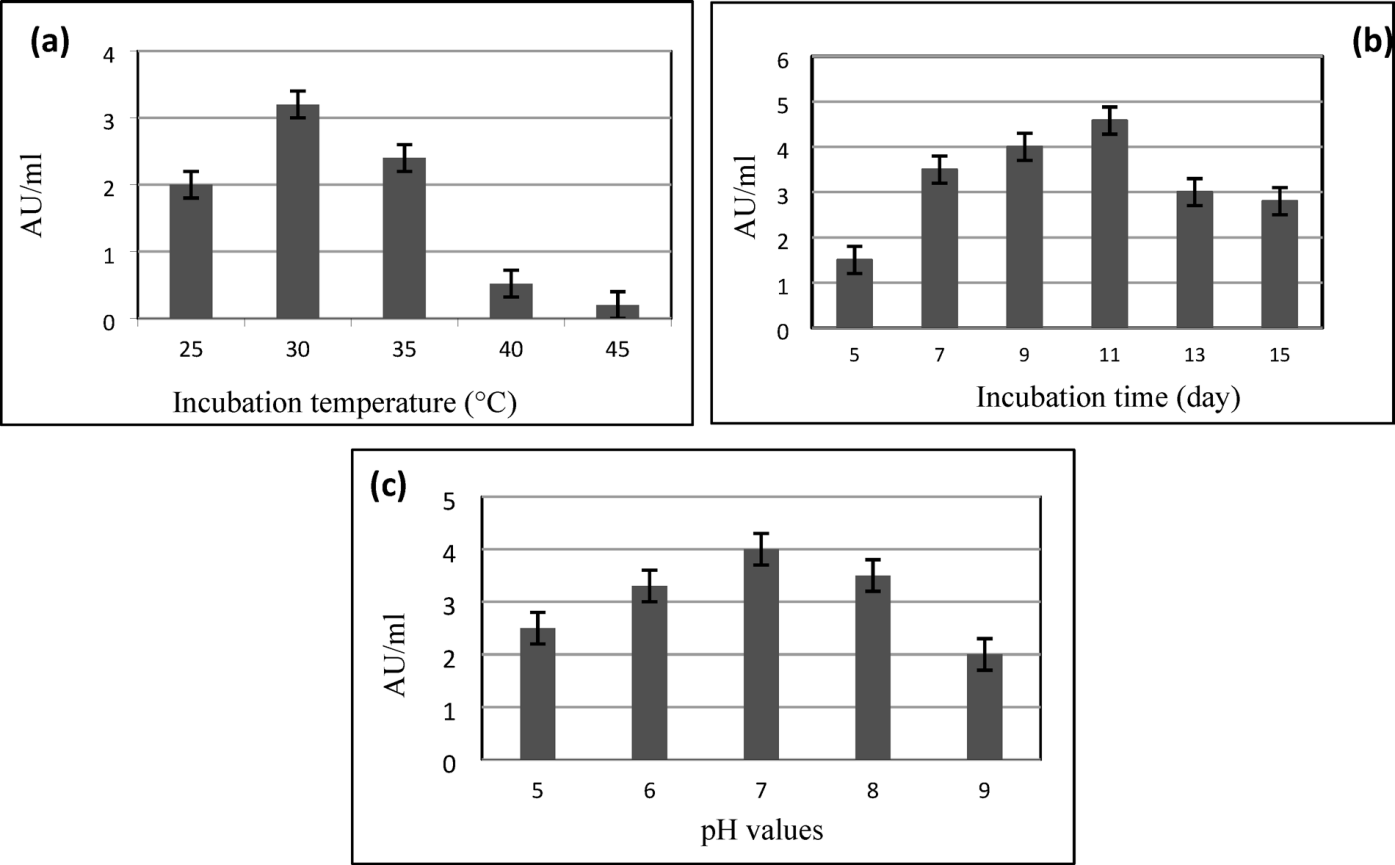
(**a**) Effect of incubation temperature, (**b**) Effect of different incubation times, and (**c**) Effect of pH on pigment production.

Methanol was used to extract the *Aspergillus turcosus PX588553* pigment, as it was reported that methanol was the most effective solubilizer of the yellow pigment^43^. The initial hints for identifying the yellow pigment are provided by the pigment’s color. The absorption spectrum analysis of the crude yellow pigment revealed that its highest absorption occurred at 430 nm^44^.The pigment’s methanol extract is collected and dried at 45° to 50 °C in a hot air oven. Microbial metabolism, in general, is significantly affected by a number of factors, including temperature, pH, and incubation time, as shown in Figs. 4(a-c).

The findings in Fig. 4-a showed that raising the incubation temperature from 25 to 30 °C enhanced pigment formation, which subsequently decreased as the temperature was raised to 45 °C. Therefore, 30 °C was the optimal temperature for the yellow pigment production. Our results agree with Abdel-Raheam et al., who reported that the maximum pigment production was at 30 °C for yellow pigment for *Monascus Ruber*^45^. According to Dikshit et al., pigment synthesis starts to decline at 35 °C^46^, and Babitha et al., found that pigment production significantly declines above 40 °C^47^.One of the most important factors of fungal pigment production is the incubation period. The results in Fig. 4-b demonstrate that the production of pigment increased progressively until it reached its highest level after 11 days, after which it gradually decreased. Our findings agree with Santos-Ebinuma et al., who reported that the production of yellow pigment by *Monascus purpureus* MTCC 410 and *Penicillium purpurogenum* DPUA1275 was achieved at 11 days^48^.Ali Abdulla et al., reported that the incubation time before 8 day produced low yields of pigment production^49^.

The pigment production is significantly influenced by the initial pH of the medium in which the organism is grown. Therefore, we created an experiment to study how different pH levels affected *Aspergillu sturcosus*a*PX588553’s* ability to produce yellow pigments.

The results in Fig. 4-c illustrated that the optimum pH for the yellow pigment production was 7.0. A pH shift above or below 7.0 led to a reduction in the amount of yellow pigments produced. Our results agree with Geweely, who reported that maximum pigment production was at pH 7.0 and 30 °C for *A. nidulans*^50^.

### Dyeing affinity towards different reaction conditions

**Fig. 5:**
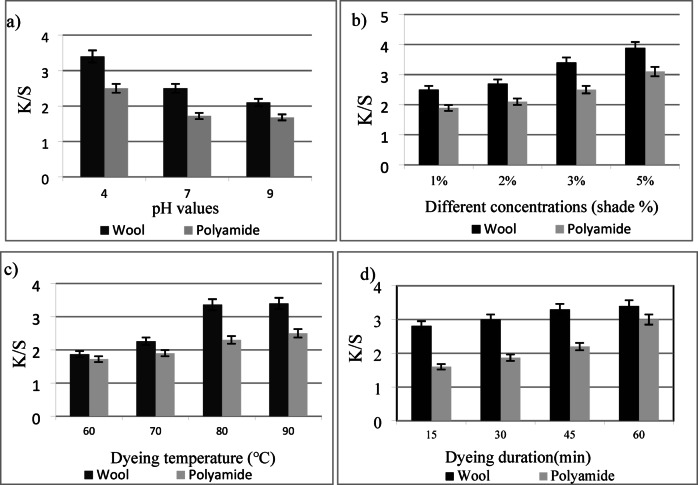
The color strength values of the dyed wool and PA6 fabrics at **a**) different pH values, **b**) different concentrations, **c**) different dyeing temperatures, and **d**) different dyeing time duration.

Wool is a biopolymer material that contains amino, carboxyl, and disulphide groups. Polyamide 6 as a synthetic polymer has a repeating amide linkage and a terminal amino group at one end of the polymer and a terminal carboxylic group at the other end in addition to its high crystallinity and limited accession to the amide groups, so based on the former facts, the effect of different reaction conditions on the dyeing process was studied and exhibited in Table 1; Figs. 5(a-b).

Table 1 showed that the dye affinity to both wool and PA6 fabric is higher at pH4, which implies that the extracted natural pigment contains chromophores or functional groups that are negatively charged (anionic). That indicates the ionic bonding interaction between the negatively charged molecules in the extracted pigment and the positively protonated amino groups in the fabrics as a result of low pH (cf. schematic mechanism 1). The fact that the dye still shows some affinity to the fabric at pH values 7 and 9 indicates that the ion-ion force is not the only way of interaction between the dye and the fabric, but there could be other interaction forces viz., dispersion forces, polar van der Waals’ forces and hydrogen bonding occurring between the hydroxyl group of extracted natural pigment and the functional groups of wool and PA6 fabrics, viz., hydroxyl, amino, or carbonyl groups, leading to textile coloration^51^.

**Scheme 1:**
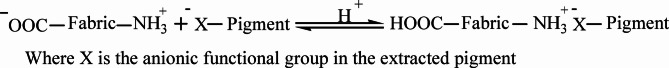
Proposed reaction mechanism between the fabric and the extracted pigment.

Table 1 showed that the increase in the dye concentration leads to an increase in the dye absorption. The colorimetric parameters such as L*, a* and b* shown in Table 1 for the dyed fabric at different concentrations and pH values. It can be noticed that the lightness (L*) value decreases in the case of dying wool and PA6 fabric with higher concentration (3% shade) and at pH 4 compared to those dyed with lower concentrations (1% and 2%) and at pH 7 and 9, and that was correlated to the high values of color strength declared in Table 1. Furthermore, the color depth is more shifted to the yellow area and less to the red area as declared by the values of a* and b*, and a high apparent depth of color occurs by increasing the dye concentration, and that is obvious by increasing the values of a* and b*, therefore, fabrics uptake more dye molecules^33^.

On the other hand, dyeing at pH 4, results in more shifting of the color depth of the dyed fabrics into the red area where the a* values are higher than those of the dyed samples at pH 7 and 9, and the b* values are also higher with dyeing at pH 4. That implies that the best dyeing conditions are dyeing with 3% shade at pH 4.

**Table 1:** Color coordinate values (L*, a*, b*) of dyed wool and PA6 fabrics with different shades and at different pH values.

Conditions	Sample	L*	a*	b*
Dyeing with 1% shade at pH 4	Wool fabric	83.9	0.85	22.9
	Polyamide 6 fabric	79.16	10.03	45.5
Dyeing with 2% shade at pH 4	Wool fabric	80.6	2.24	32.14
	Polyamide 6 fabric	76.16	10.43	46.4
Dyeing with 3% shade at pH 4	Wool fabric	73.5	5.5	42.7
	Polyamide 6 fabric	72.2	11.4	48.2
Dyeing with 5% shade at pH 4	Wool fabric	65.5	6.3	45.1
	Polyamide 6 fabric	63.2	12.5	50.2
Dyeing at pH 4 with 3% shade	Wool fabric	73.5	5.5	42.7
	Polyamide 6 fabric	72.2	11.4	48.2
Dyeing at pH 7 with 3% shade	Wool fabric	80.14	-1.51	19.19
	Polyamide 6 fabric	75.16	-1.51	19.19
Dyeing at pH 9 with 3% shade	Wool fabric	81.77	-0.64	27.07
	Polyamide 6 fabric	77.16	-1.3	20.58

The effect of dyeing temperature and time was also studied, and the data from Figs. 5(a-d) reveals that the color strength values of the dyed fabric are almost comparable by dyeing at temperatures 80 °C and 90 °C, moreover that data after dyeing for 30 min is also comparable to those after dyeing for 45 and 60 min for wool fabrics. So it can be concluded that the optimum conditions for dyeing are at 3% dye shade, at pH 4, at 80 °C for 30 min for wool fabrics and 60 min for polyamide 6 fabrics, with L.R. 1:50.

### Mordant effect

Table 2 showed the color strength data and CIE Lab colorimetric coordinates of the dyed fabrics after using mordant. Data from the table shows a slight increase in the color strength in case of dying wool fabric compared to the dyed fabrics without mordant. Data also declares a higher shift in the color depth in the case of dyeing wool fabric using mordant, indicated by the L*, a* and b* values. These data showed that using mordant caused a hyper-chromic shift and increased the depth of the color. So, in addition to ionic bonding, the dye affinity to fabrics was dependent on the formation of a metal complex between dye, fiber and metal (mordant). Therefore, by applying metal salt, the dye uptake and the washing fastness are increased (cf. Table 3)^52^.

On the contrary, color strength of the dyed PA6 fabrics decreased in addition to the slight shift in color depth declared by L*, a* and b* values that means it caused a hypo-chromic shift, and that could be attributed to the addition of metal mordants, which formed metal–dye complexes, and the complexes may interact differently with the terminal protonated amine sites on PA6 fibers, reducing effective dye–fiber substantively associated with the free dye molecules (*c.f*. Schematic mechanism 2) i.e., both the formed complex and sulphate anions of the mordant occupy the same active sites needed for dye adsorption^34^, which also affected the washing fastness as shown in Table 3. Finally, the low modified RUI values (< 0.2) confirm uniform levelness of the dyed samples, suggesting negligible variation in color strength across different spots of the fabric.

**Fig. 6:**
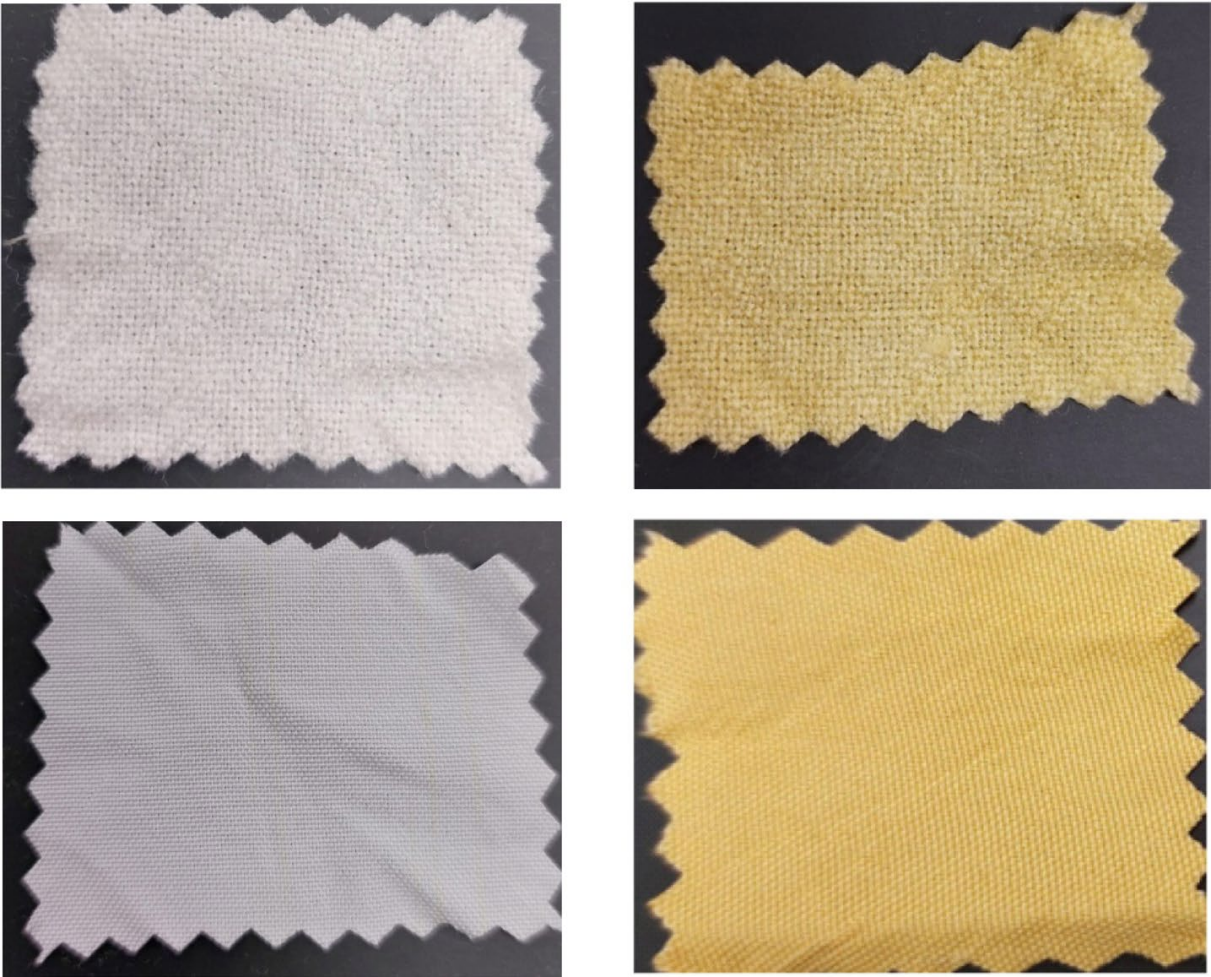
comparative photographs for the blank and dyed wool fabrics in the upper side and blank and Dyed PA6 on the lower side.

Sharma et al. have isolated three fungi, namely *Trichoderma virens*, *Alternaria alternata* and *Curvularia lunata*,to get pigments for textile dyeing, and the color data was comparable to the data in the present study(with the used concentrations which can be increased and hence the color data will be increased as well)^53^. Also, an isolated strain of *Vibrio* sp. from marine sediments produced red pigments that could be used to dye many fibers, including wool, nylon, with K/S data ranging from 3.2 to 3.4 and the results also showed a 15% reduction in concentration in the solution after 60 min of the treatment, which reveals that the colorant is not highly stable in acidic solution at elevated temperature, unlike the extracted pigment in the present study^54^. Photographs for the fabrics before and after dyeing using the optimum conditions were represented in Fig. 6.

**Table 2:**
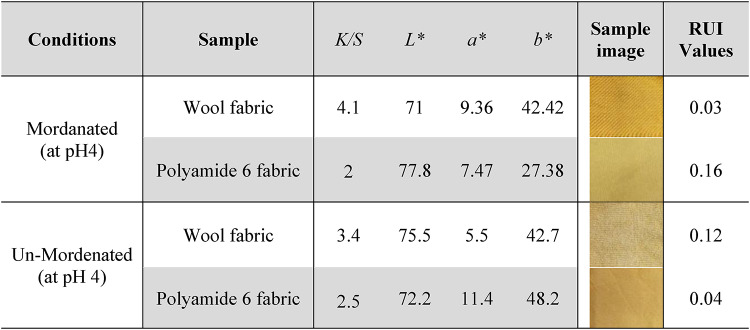
Color coordinate values (L*, a*, b*) of dyed wool and PA6 fabrics with and without using mordant.

**Scheme 2:**
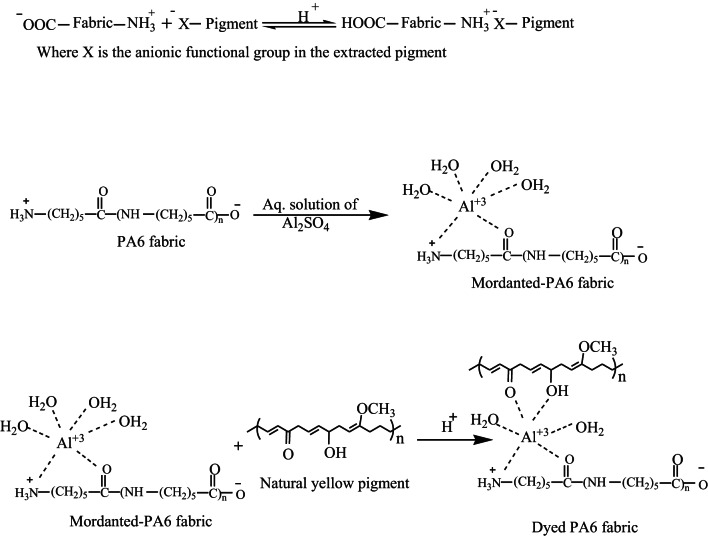
Coordination interaction between metal mordant, nylon 6 fabric and extracted pigment (as suggested from HNMR and FTIR analyses).

### Fastness properties

Data of Table 3 shows the washing and light fastness of the dyed fabrics at different pH and in the presence and absence of mordant. The data in the table shows a variation in the washing and light fastness with pH change for the dyed samples. At pH 4 protonation of amino groups on wool and PA6 fabrics enhances electrostatic attraction with anionic functional groups present in the extracted natural pigment strengthening the bonding between the fabric and dye molecules, in addition to H-bonding and metal-dye-fiber comploxation. That was obvious from the data; mordanted dyed wool fabric showed good washing fastness while dyed wool fabric without using mordants gained lower values of fastness. In contrast, PA6 fabrics exhibited improved washing fastness, in the absence of mordant, and that was discussed in the previous section (Sect. 3.5, **Mechanism 2**), where the ionic interaction with the protonated amino groups and the H-bonding are sufficient interactions to ensure good dye fixation without the need for a mordant. It is worth mentioning that samples show relative improve in light fastness results at pH 4 for several reasons viz., the functional groups and conjugated system in the extracted pigment allowed stronger binding in addition to partial stabilization of the chromophore in moreover that, coordination complexes formation by mordant which lead to improving their resistance to photodegradation^33^. Light fastness is administered by photochemical reaction; it is correlated to the attributes of the chromophores present in the pigment, and it improves by increasing the depth of color^55^. The data suggests that the dyed fabrics are more predominant for the indoor textile products and not to those exposed to sunlight. The dyed fabrics at pH 4 also exhibited rubbing fastness ranging from 4 to 4–5. The adequate rubbing fastness values designate that there are limited surface aggregations of the extracted pigment and suggest that most of the molecules were effectively fixed within the fabric matrix, not on the surface, which is consistent with the high washing fastness results. It can be concluded that the main chemical interaction between the dye and the fabric took place via hydrogen bonding, and ionic interaction and by using mordant, dye molecules interact via complex formation.

**Table 3:** Fastness properties of wool and PA6 fabrics dyed at different reaction conditions

Conditions	Sample	Washing			Rubbing		Light fastness
		Staining		Alt.	Dry	Wet	
		St*	St**				
Mordanated (pH4)	Wool fabric	4-5	4-5	4	4-5	4	4
	Polyamide 6 fabric	4	4	4	4	4	3-4
Un-Mordenated (pH 4)	Wool fabric	4	4-5	4	4	4	3-4
	Polyamide 6 fabric	4-5	4-5	4	4-5	4	3-4
Dyeing at pH 7	Wool fabric	4	4	3	3-4	3-4	2-3
	Polyamide 6 fabric	3-4	3-4	3	3-4	3	2-3
Dyeing at pH 9	Wool fabric	3-4	3-4	3	3	3	3
	Polyamide 6 fabric	3	3	3	3	3	2-3
st* = staining on cotton, st** = staining on wool, Alt. = Alteration in color							

In general it can be noticed from the color data and the fastness properties that wool fabrics provide a better color strength data and better fastness than do polyamide 6 fabrics. This can be attributed to several factors that characterize wool fabric over PA6, viz., the chemical nature of wool fabrics that based on protein structure which is rich with amino and hydroxyl groups that offers more active sites for natural dyes. Unlike PA6 which is synrthetic fabric has amide linkages in addition to terminal amino and hydroxyl groups that provides lower reactive sites for dye access. Moreover that, the morphological structure of both fabrics interfere their reactivity and dye uptake. The scaly structure of wool fabric controls the dye uptake by controlling the diffusion pathways through intercellular gaps^56^ an obviously that feature is absent in the smooth structure of PA6 leading to different dyeing and surface interaction behavior.

### Halochromic test

The CIE Lab colorimetric coordinates of the fabrics were consequently evaluated and are exhibited in Table 4.The data of ∆E indicates the sensitivity of the extracted yellow pigment to pH changes. It can be seen that at pH 4, the samples become darker as the L* values decrease; moreover, a* and b*prove that the colour of the fabrics changed from yellowish to reddish (a*evidences higher values, and b* has a sharp decline). The larger ∆E values, along with the significant changes in a* and b*, indicate the noticeable colour shift by the naked eye and not only lightness change.

**Table 4:**
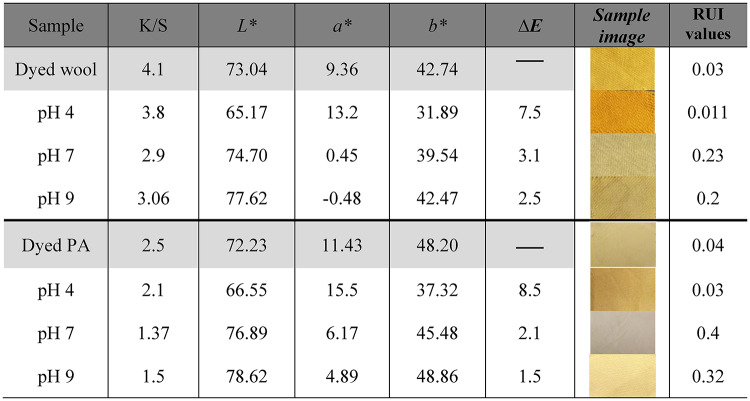
Color strength values and color coordinate values (L*, a*, b*) of dyed wool and PA6 fabrics after immersion in buffered solutions at pH 4, 7 and 9.

### Nuclear magnetic resonance (NMR)

**Fig. 7:**
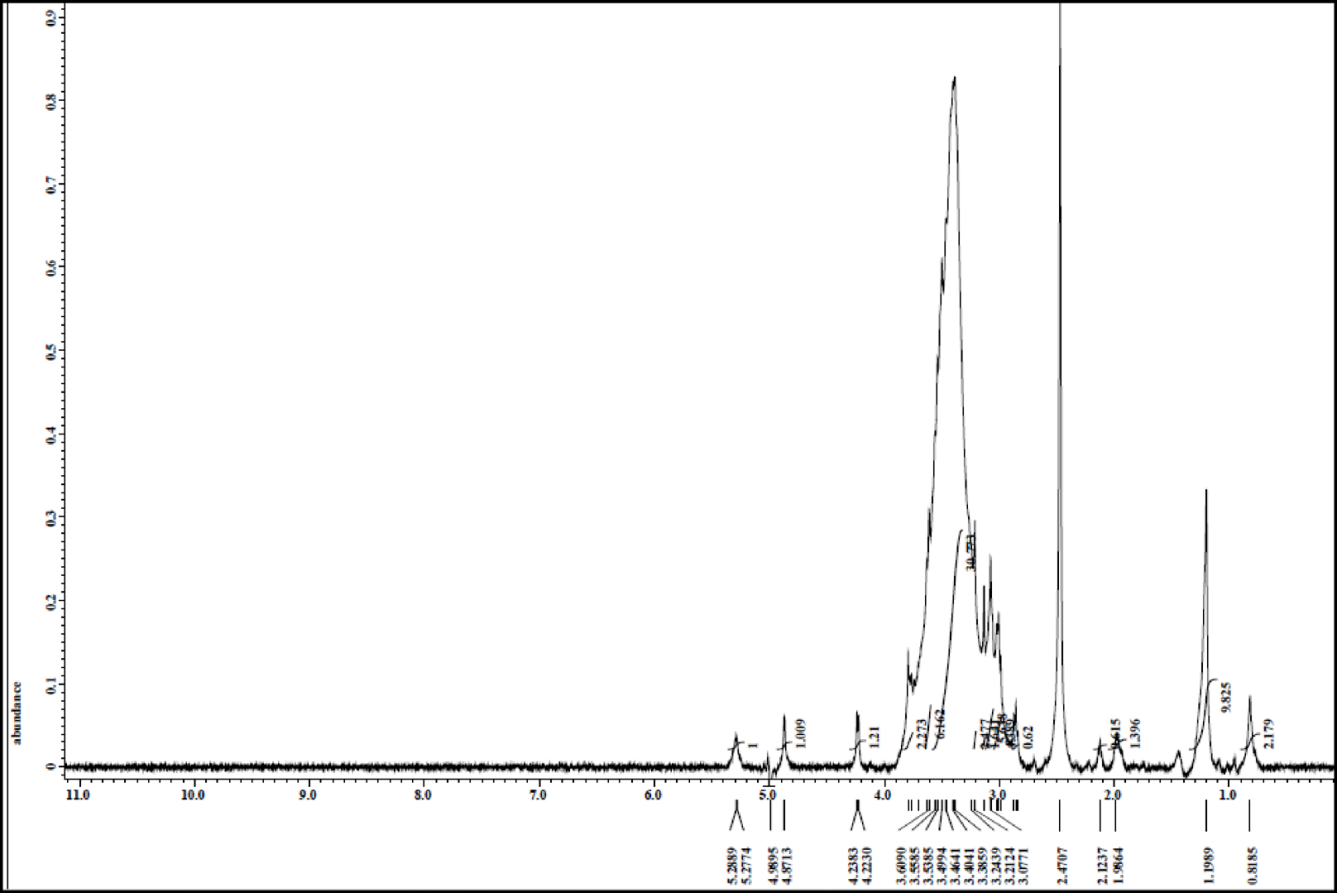
^1^HNMR spectra of the extracted yellow pigment.

In our study, we examine the ^1^HNMR spectra of the yellow pigment (Fig. 7). We found that the ^1^HNMR spectra at signals 5.28 and 4.22 ppm detected the presence of conjugated double bonds and at signal 3.60–3.07 ppm detected methoxy groups or hydroxylated aliphatic chains. Carbonyl-adjacent protons exhibited a signal at 2.47 ppm. The hydrocarbon side chain at 1.20–0.82 ppm indicates the presence of aliphatic methyl or methylene protons in saturated aliphatic chains. A signal between 2.1 and 1.98 ppm was assigned for methylene groups or allylic groups. This data is commonly detected in yellow fungal pigment^57,58^.

### Fourier transform infrared spectroscopy (FTIR)

**Fig. 8:**
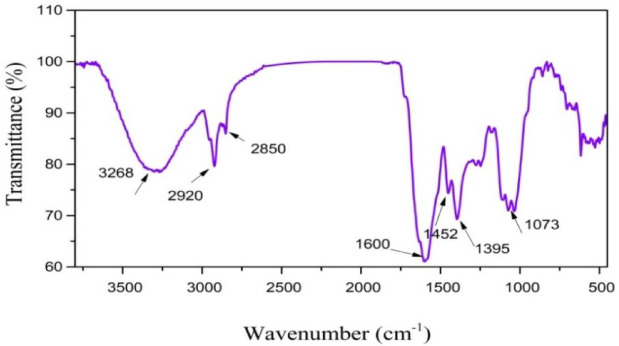
The FTIR chart of the extracted natural dye.

Beginning with the IR chart of the extracted pigment (cf. Figure 8), several bands can be noticed in the chart that can be interpreted as follows:

The band at 3267 cm^− 1^ corresponds to O-H stretching of alcohol; the bands at 2920 and 2850 cm^−1^correspond to stretching vibration of symmetrical and asymmetrical stretching of –CH_2_, and –CH_3_ groups; the bands at 1599 and 1452 cm^− 1^ correspond to double bond (C = C) stretching vibrations and C–H bending of conjugated ring; and the band at 1395 cm^− 1^ is due to the O–H bending of alcohol. Finally, the stretching vibration band at 1073 cm^−1^corresponds to the C-O of primary alcohol^59^. Additionally, in complex natural pigments, the C = O stretching vibration may shift to lower wave numbers and overlap with C = C vibrations around 1600–1650 cm⁻¹, resulting in a broadened or merged band. Also the intra- and intermolecular H-bonding weaken and broaden the C = O band.

Wool and polyamide have well-defined characteristic IR bands due to their repeating units, so new functional groups within the fabric are not highly expected. The natural dyes primarily interact through intermolecular forces or through forming relatively weak bonds that do not change the primary chemical structure of wool and polyamide.

**Fig. 9:**
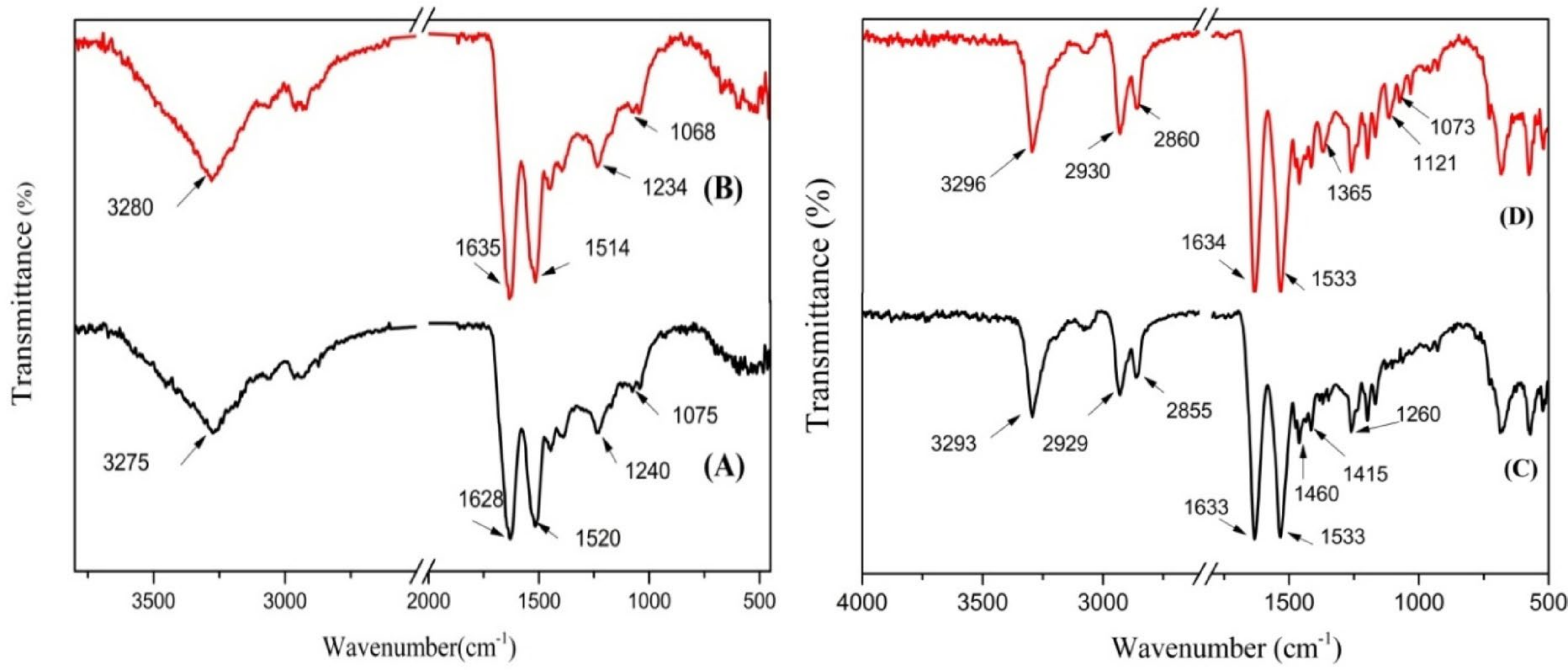
The FTIR chart of **A**) Blank wool, **B**) Dyed wool, **C**) Blank PA6, and **D**) Dyed PA6 fabrics.

Figure 9(A-B) shows the FTIR chart of the native and dyed wool fabric with the extracted natural pigment in the presence of mordant. The chart of native fabric shows the usual bands of wool at 3275, 1628, 1520, 1240 and 1075 cm^− 1^ which corresponds to O-H stretching, amide I connected to C = O stretching vibration, amide II of N-H bending vibration, amide III band due to the C − N stretching and N − H in-plane bending vibrations, and the stretching vibration band of C-O of primary alcohol, respectively^60^. The IR shift in the bands’ position and intensity is observed in the chart of the dyed wool fabric, which indicates the change in the vibration energy levels of the chemical bonds formed by the interaction of the fabric with the molecules of the natural dye.

That chemical interaction may involve hydrogen bonding, ionic bonding or covalent bonding; this interaction changes the electron density of the bonds and consequently the strength of the existing bonds in the fabric. The change in the bond’s strength leads to shifts in the IR bands^61^.It is worth mentioning that the low shift in the IR bands is an indication for the hydrogen bond formation.

The shift in the bands may also take place as a result of mordanting the samples with aluminum sulphate followed by dyeing^62^.

Figure 9(C-D) shows the characteristic bands of the FTIR spectrum of PA6. It can be noticed that bands at ~ 3290 cm^− 1^ and 1533 cm^− 1^ were assigned to the N-H stretching and bending vibration band (amide II)^63^, also the carbonyl stretching vibration band at ~ 1633 cm^− 1^ (amide I). In addition to that, the band at 1260 cm^− 1^ which corresponds to the C-N stretching band and the bands at ~ 2929, 2855, 1461 and 1415 cm^− 1^ are assigned to the vibration of symmetrical and asymmetrical stretching of –CH_2_, and–CH_3_ groups,–CH_2_– scissor and bending vibration, respectively. And the chart data is approved to the literature spectrum of PA6^64^.

Figure 9-D exhibits the FTIR chart of the dyed PA6 fabric. It can be noticed that, in addition to the characteristic peaks of PA6 fabric, new bands are formed at 1365, 1121 and 1073 cm^− 1^. These bands can be assigned as follows: the band at 1365and 1073 cm^− 1^ could be correlated to the O-H bending and C-O of alcohol, respectively and these bands were observed in the FTIR chart of the extracted natural dye. Even if the OH stretching band is not visible in the FTIR chart of dyed PA6, the dye is clearly on the fabric, and its O-H and C-O will appear in the spectrum. The band at 1121 cm^− 1^ could be attributed to ether linkage formed by the reaction between the hydroxide group of the dye and the terminal groups of PA6, viz., -COOH, -NH_2_ or even one of the reactive sites of PA6 forming either ester or ether linkages, which has C-O stretches in the region 1070–1150 cm^− 1^.

The missing OH broadband in the dyed PA is may be due to the reaction between the dye molecules and the fabric via hydroxyl groups, forming new hydrogen bonds with the functional groups (-NH, -C = O); the change in the hydrogen network can lead to a change in the intensity and decrease the broadening of the band^65^. Moreover the concentration of the dye that used to dye the fabric might be relatively small compared to the total mass analysed in the FTIR spectrometer, so if the concentration of the –OH groups from the dye on the fabric is low, the resulting -OH stretching band may be weak and difficult to distinguish or even overlap with the –NH stretching band present in wool and polyamide. This overlap makes the –OH band of the dye diminish or merge into the –NH band.

### UV-Vis spectroscopy

**Fig. 10:**
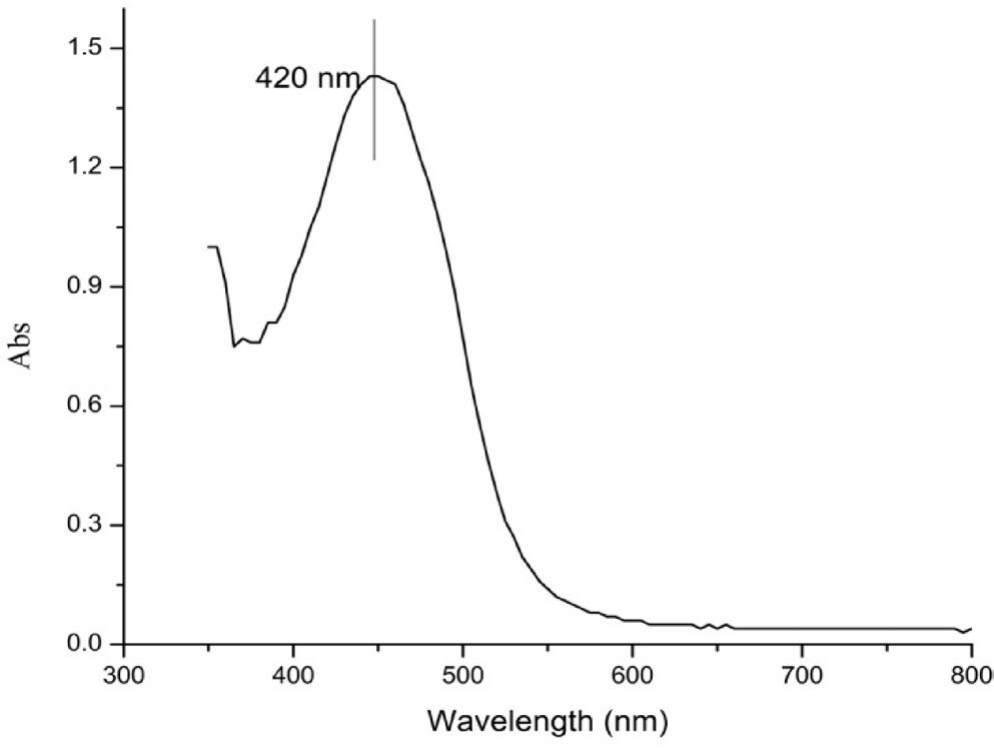
UV-Vis spectra of the extracted yellow pigment.

The UV–Vis spectrum of the extracted pigment Shown in Fig. 10 exhibits a maximum absorption peak at λ_max_ 420 nm in the visible region, responsible for its characteristic yellow color. The broad band represents π–π* electronic transitions of conjugated system which is typical for natural dyes. The broad absorption band extends into the UV region approximately 280–400 nm covering both UVB and UVA ranges, suggests the presence of conjugated chromophores capable of UV absorption.

The FTIR spectrum showed absorption bands at 1599 and 1452 cm^-1^ correspond to double bond (C = C) stretching vibrations and C–H bending of conjugated ring; while the ¹H NMR spectrum showed spectra at signals 5.28 and 4.22 ppm detected the presence of conjugated double bonds. Additionally the UV–Vis absorption indicates extended conjugation of the chromophoric groups, along with FTIR and NMR data suggesting that the pigment consists of a highly conjugated system.

### X-ray diffraction pattern

**Fig. 11:**
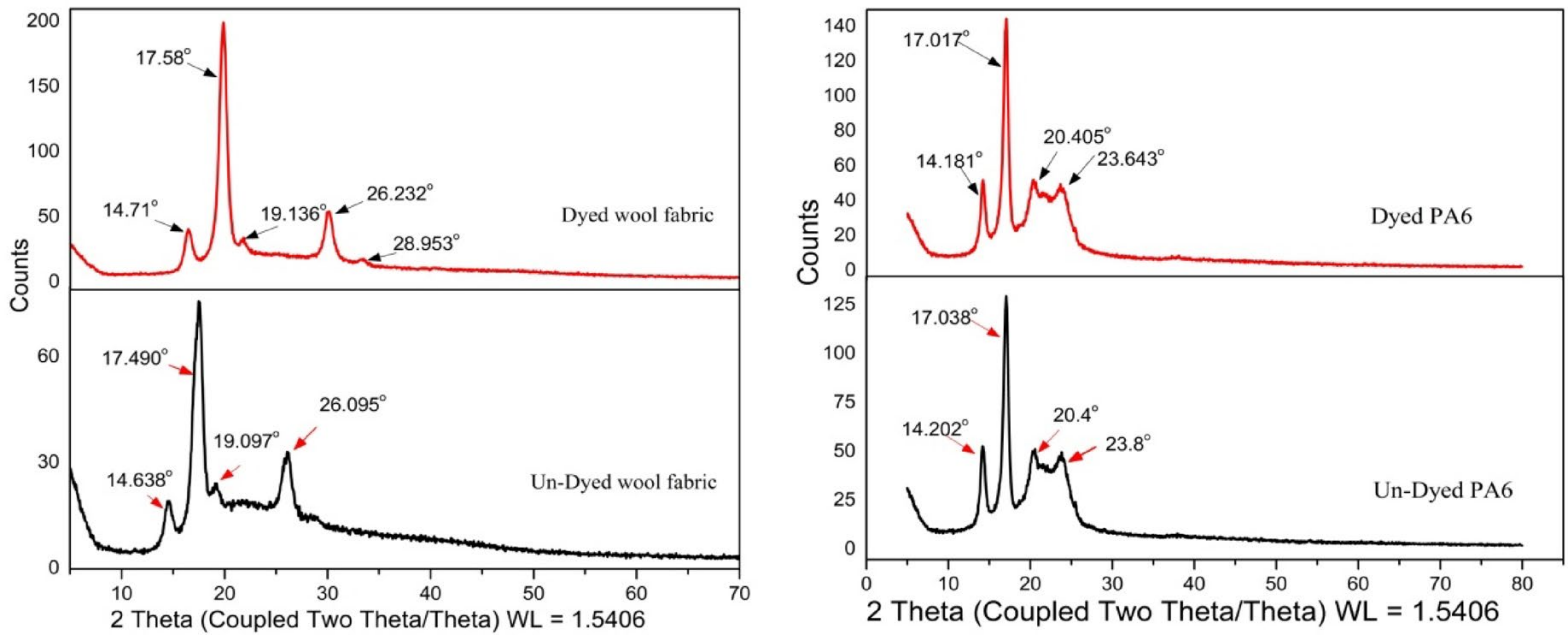
The XRD diffractograms of blank and dyed wool fabric on the left side and blank and dyed PA6 on the right side.

The crystalline structure of PA6 fabric is affected by the presence of hydrogen bonding between amide and carbonyl groups. According to H-bonds, there are two different forms of the crystalline phases: α-phase and γ-phases. The strength of the H–bond in the α-phase is higher than in the γ-phase^66^. Figure 11 shows the XRD pattern of undyed and dyed PA6 fabric, respectively. The spectrum shows two characteristic peaks at 2θ = 20.4° and 23.8° assigned for the α-phase structure. An unremarkable shift in the positions of the diffraction peaks was measured from the XRD spectrum of PA fabric after dyeing with the extracted natural dye. In addition to that Table 6 revealed a slight increase in the peak intensity of the dyed PA6 compared to the undyed sample; the increases in the intensity of the peaks signify a change in the crystalline structure (i.e., an increase in the α-phase structure)^67^. This implies that some amorphous or disordered regions in the PA6 structure have transformed into crystalline regions this is due to dyeing conditions at elevated temperatures in aqueous solution. The dye molecules interact with PA6 fabric via H-bonding, covalent bonding or even minor ionic interaction that might make the dye molecules penetrate into the chains of PA6.The hydroxyl group from the dye may form a covalent bond with PA6 chains, forming an ether linkage; that finding is in agreement with the data of the FTIR chart of PA6^68^.

Figure 11 shows the XRD spectrum of wool that shows characteristic peaks at 2θ = 14.6° corresponding to the amorphous nature of wool and 2θ = 17.5°, 19.1° and 26.1°, which are assigned to the β-sheet like structure of wool fabric^69^. Like in the case of dyed PA6, the spectrum the dyed wool fabric shows a small shift in the positions of the diffraction peaks. Moreover the intensity of the peaks was changed as shown in Table 5, where the peak intensities were highly increased compared to their intensities in the spectrum of un-dyed wool fabric, which emphasized the increase in the crystalline structure of wool fabric after dyeing with the extracted natural dye^70^. The diffraction spectrum of the dyed wool fabric displays a new peak at 2θ = 28.9°.This might be explained by the dyeing process causing significant swelling and disruption in the main crystalline structure of wool by breaking the intermolecular hydrogen bonding within the wool side chains. This disruption in hydrogen bonds caused a decrease in the density of hydrogen bonds in the native crystalline structure that made the chains of wool more free to move and that may cause a rearrangement in the chains into a new crystalline phase that gives a new peak^71^.

**Table 5:** Relative intensities and crystallinity indexes of dyed and undyedwool and PA6 fabrics.

Sample	2θ	d-spacing (A°)	Net intensity	CI %
Blank wool fabric	14.638°	6.04660	320.520	60
	17.490 °	5.06664	2061.35	
	19.097°	4.64373	307.910	
	26.095°	3.41209	577.621	
Dyed wool fabric	14.71°	6.01741	901.820	70
	17.58 °	5.04031	6135.72	
	19.136°	4.63418	445.345	
	26.232°	3.39455	1223.19	
	28.953°	3.08139	109.528	
Blank PA6 fabric	14.202°	6.23108	1218.71	50
	17.038°	5.20001	3738.07	
	20.4°	4.33974	1045.96	
	23.8°	3.73060	1138.07	
Dyed PA6 fabric	14.181°	6.24039	1219.40	55
	17.017°	5.20621	4255.30	
	20.405°	4.34880	1072.92	
	23.643°	3.76005	1101.88	

### Ultraviolet protection factor

UV radiation coming from the sun is composed of UV-A (400–315 nm), UV-B (315–290 nm) and UV-C (below 290 nm). UV-C is absorbed by the ozone layer, but UV-A and UV-B reach the earth, causing hazards for human and animal health. So, UV-protective textile is needed and is represented in UV transmittance and UV protection factor. UV-protective textiles were accomplished before by different ways, viz., using different structures of the textile material^72^ or by chemical treatments .

The results shown in Table 6 of UPF of the dyed wool fabrics are categorized as very good UV-protection, while that of PA6 is good UV-protection according to standard values of AS/NZS 4399:1996 for textile shielding. The results of UPF, UV-A and UV-B confirm the protection of the dyed fabrics against UV radiation; and this can be attributed to the UV-absorbing ability of the conjugated chromophores of the yellow pigment extract, proved by FTIR, and ^1^HNMR, in addition to the UV–Vis absorption spectrum. These conjugated chromophoric groups capable of effectively absorbing harmful UV radiation. Hence applying the yellow pigment to the fabrics acts as UV absorbers, this reduces UV transmission through the textile structure leading to enhanced UPF values.

The UPF results are consistent with previously reported data and demonstrate the effectiveness of the extracted natural pigment in enhancing UV protection^73^.

**Table 6:** UV protection characteristics of the untreated as well as dyed wool and PA6 fabric.

Sample	UPF value	T(UV-A)	T(UV-B)
Blank wool fabric	12	20.8	14.5
Dyed wool fabric	35	5.3	7.7
Blank PA6 fabric	2	37.5	44.8
Dyed PA6 fabric	15	6.5	22.5

### Antimicrobial activity of yellow pigment and the dyed fabrics

Using natural microbial dyes with antibacterial effects to make medical textiles, such as wound dressings, and to create clothing for babies and people allergic to synthetic dyes is an interesting challenge^74^. Antibacterial activity of the dyed textile (polyamide and wool) and yellow pigment was examined in this investigation. According to the results in Table 7, the yellow pigment and dyed textile (wool and polyamide) showed antibacterial activity against gram-negative bacteria (*Escherichia coli* NRRL-B210) and gram-positive bacteria (*Bacillus subtilis* NRRL-B543).These findings demonstrated that the yellow pigment’s antibacterial properties could be maintained after it was bonded to the fabric. The antibacterial activity of the yellow extracted pigment was attributed to its chemical structure, where the -OH and –OCH_3_groups permit the H-bonding with the bacterial cell membrane, which cause an increase in its permeability and consequently leads to cell membrane damage^75^. Additionally, the conjugated system of the extracted pigment disrupts the lipid bilayer of the bacterial cell and also enhances the bactericidal effect by inhibiting intracellular DNA gyrase^76^. The effect of the carbonyl group can also be attributed as carbonyl compounds readily react with nucleophilic groups such as thiol and amino groups on the bacterial proteins, causing protein disruption in addition to their reaction with the bacterial cell wall and cytoplasmic membrane, increasing their permeability and hence causing direct damage to them^77^. Our results agree with Abou Elmaaty et al., who reported that yellow pigment-dyed wool and polyester have excellent antibacterial activity against gram-positive and gram-negative bacteria^74^. The yellow-dyed polyamide fabric showed greater antibacterial activity than dyed wool; this may be due to the polyamide’s hydrophobic surface maintaining the pigment’s superior efficacy against bacterial strains. On the other hand, wool’s porous, protein-based structure may allow the pigment to be absorbed deeper into the fibers, reducing its surface antibacterial activity. This suggests that the type of fiber has a significant effect on the bioactivity of textiles treated with pigment^78^.The ability to withstand washing must be a feature of all textiles; therefore, the wool and polyamide fabrics stained with yellow pigment were washed 20 times, and their antibacterial activities were evaluated. The results showed that the dyed fabrics still have antibacterial activity against *Escherichia coli* NRRL-B210 and *Bacillus subtilis* NRRL-B543.

Hence, it can be concluded that the presence of oxygen-containing functional groups like hydroxyl, carbonyl, and methoxy moieties in these pigments causes them to exhibit biological activity.

In the previous studies, optimization of bacterial and fungal dye from the environment was extracted and applied to different types of fabrics and showed bacterial inhibition zones ranging from 5 to 15 mm for different types of pathogenic bacteria, which is less effective than the extracted pigment in the present study, according to the data shown in Table 7^22^. Also, the inhibition zone of natural dye pomegranate-treated wool substrates against *S. aureus and E. coli* was reported to range from 8 to 3 mm, respectively^79^.

**Table 7:** The antibacterial activity of the extracted yellow pigment, native and dyed wool and PA6 fabrics against *Bacillus subtilis* NRRL-B543 and *Escherichia coli* NRRL-B210.

Sample	Inhibition zone diameter (mm) against tested bacterial strains	
	B.subtilis	E. coli
Extracted yellow pigment	42	45
Untreated wool	--	--
Dyed wool fabric	25	21
Mordanted-Dyed wool fabric	31	36
Mordanted-Dyed wool fabric after 20-washing cycle	28	27
Untreated PA6	--	--
Dyed PA6 fabric	27	30
Mordanted-Dyed PA6 fabric	35	37
Mordanted-Dyed PA6 fabric after 20-washing cycle	24	25

### Antioxidant activity of yellow pigment extract

**Fig. 12:**
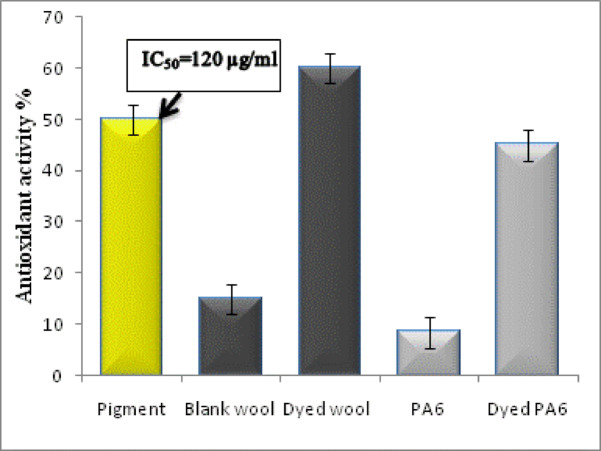
Antioxidant activity of the extracted pigment blank and dyed wool and PA6.

The results in Fig. 12 showed that as the concentration of the yellow pigment increased, its ability to combat DPPH radicals also increased, suggesting that it is a potent antioxidant. At 50 µg/mL, the inhibition was 31.1%; at 100 µg/mL, it was 42.2%, and by increasing the pigment concentration, the antioxidant inhibition was found to be increased, and its IC_50_ reached to 120 µg/mL, suggesting that the pigment contains functional groups that can neutralize free radicals by fabric-pigment bonding. This activity is most likely caused by the conjugated double bond, and hydroxyl, systems in the pigment structure, as determined by FTIR and ¹H NMR spectroscopy^58^. Wool showed higher DPPH inhibition (60%) than polyamide (45%). This is due to the binding of hydroxyl-rich pigments to the wool proteinaceous keratin structure that may have caused the stronger activity on wool, increasing the availability of antioxidant functional groups on the fiber surface^80^. On the contrary, blank control fabrics showed less activity, confirming that the yellow pigment itself is the source of the antioxidant action. The yellow pigment is bioactive when it is in solution form or when applied to textiles. The antioxidant activity was found to be consistent with the published data of fabrics dyed with different natural dyes in literature^16,81^.

## Conclusion

The integration, of natural dyes with inherent antibacterial and antioxidant properties into wool and polyamide 6 fabrics, applies different textile applications in different sectors. Wool and polyamide fabrics were dyed with the pigment extracted from *AspergillusturcosusPX588553*,and the colorimetric data confirmed the affinity of wool and polyamide 6 fabrics to the extracted yellow pigment. The dyed fabrics showed enhanced UPF and a good resistance to the harmful UV radiation. The chemical analyses, viz., NMR and FTIR, proved and suggested the type of chemical bonding between the extracted pigment with wool and PA6 to be basically hydrogen bonding and ionic interaction in addition to interactions between dye and the fabric by using mordant through complex formation.

The antibacterial activity and antioxidant properties of the dyed fabrics showed a great enhancement compared to the native wool and PA6 fabrics even after about 20 washing cycles, which proves the incorporation of the extracted pigment with the fabrics chemically. The enhanced antibacterial, antioxidant and dyed fabrics were suggested for many applications, viz., functional apparels for everyday clothing (e.g., socks and active wear) that actively protect the skin from bacterial growth, odor and environmental free radicals, helping to reduce inflammation and help healing by neutralizing the free radicals, in addition to children’s and baby clothing. Moreover, the hospitals and medical uniforms and applications e.g., gowns, scrubs, patient gowns and bed sheets, besides wound dressings and bandages.

## Supplementary Information

Below is the link to the electronic supplementary material.

Supplementary Material 1

Supplementary Material 4

Supplementary Material 3

Supplementary Material 2

## Data Availability

The authors confirm that the data supporting the findings of this study are available within the article.
