# Supplementary material for: A green approach to antibacterial and antioxidant wool and polyamide 6 fabrics through bioactive Aspergillus turcosus extracted pigment for healthy and high-performance textile products
Source: Sci Rep. 2026 Jun 19;16:19168. doi: 10.1038/s41598-026-55888-w (PMC13282385; doi:10.1038/s41598-026-55888-w)
Supplement: Supplementary file 1 — Supplementary Material 1 [file 41598_2026_55888_MOESM1_ESM.docx]

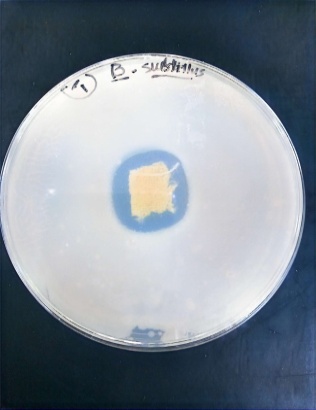

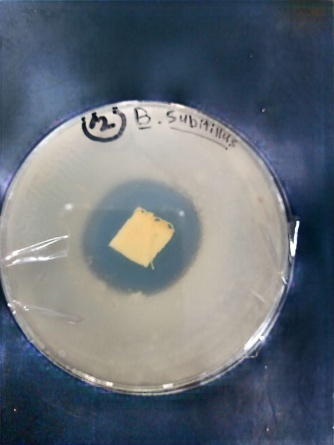


**d)**

**c)**

**b)**

**a)**

**e)**

**f)**


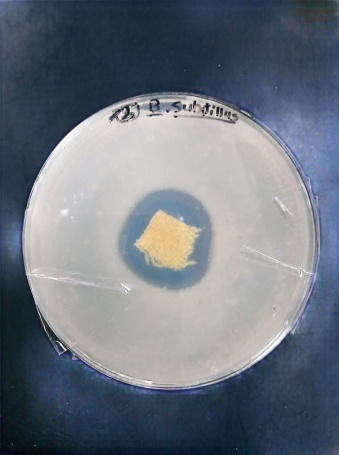

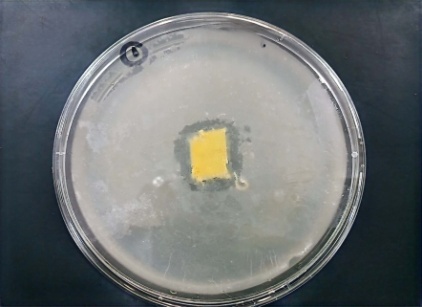


**a)**

**S1:**The inhibition zones of gram-positive bacterial growth of a) extracted pigment, blank and dyed wool fabric, b) extracted pigment, blank and dyed PA6 fabric, c) mordanted-dyed wool fabric, d)mordanted-dyed PA6, e) mordanted-dyed wool fabric after 20 washing cycle, f) mordanted-dyed PA6 fabric after 20 washing cycle, incubated with *Bacillus subtilis* NRRL-B543 respectively.


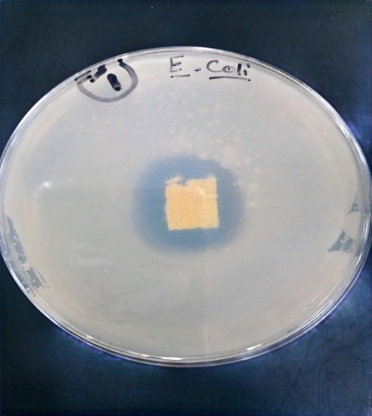

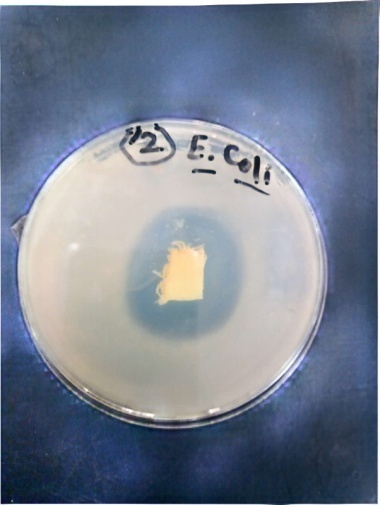


**l)**

**k)**

**h)**

**g)**

**j)**

**i)**


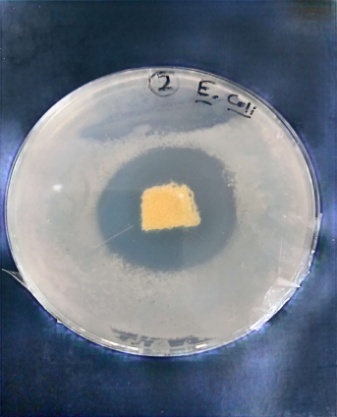

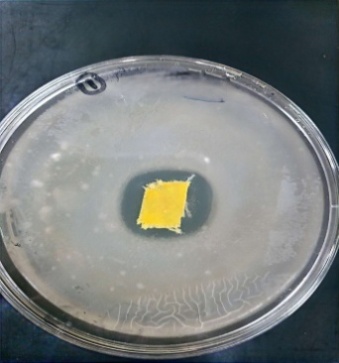


**S2:**The inhibition zones of gram-negative bacterial growth g) extracted pigment, blank and dyed wool fabric, h) extracted pigment, blank and dyed PA6 fabric, i) mordanted-dyed wool fabric, j)mordanted-dyed PA6, k) mordanted-dyed wool fabric after 20 washing cycle, l) mordanted-dyed PA6 fabric after 20 washing cycle, incubated with *Escherichia coli* NRRL-B210 respectively.
