# Supplementary figures and images for: A green approach to antibacterial and antioxidant wool and polyamide 6 fabrics through bioactive Aspergillus turcosus extracted pigment for healthy and high-performance textile products
Source: Sci Rep. 2026 Jun 19;16:19168. doi: 10.1038/s41598-026-55888-w (PMC13282385; doi:10.1038/s41598-026-55888-w)

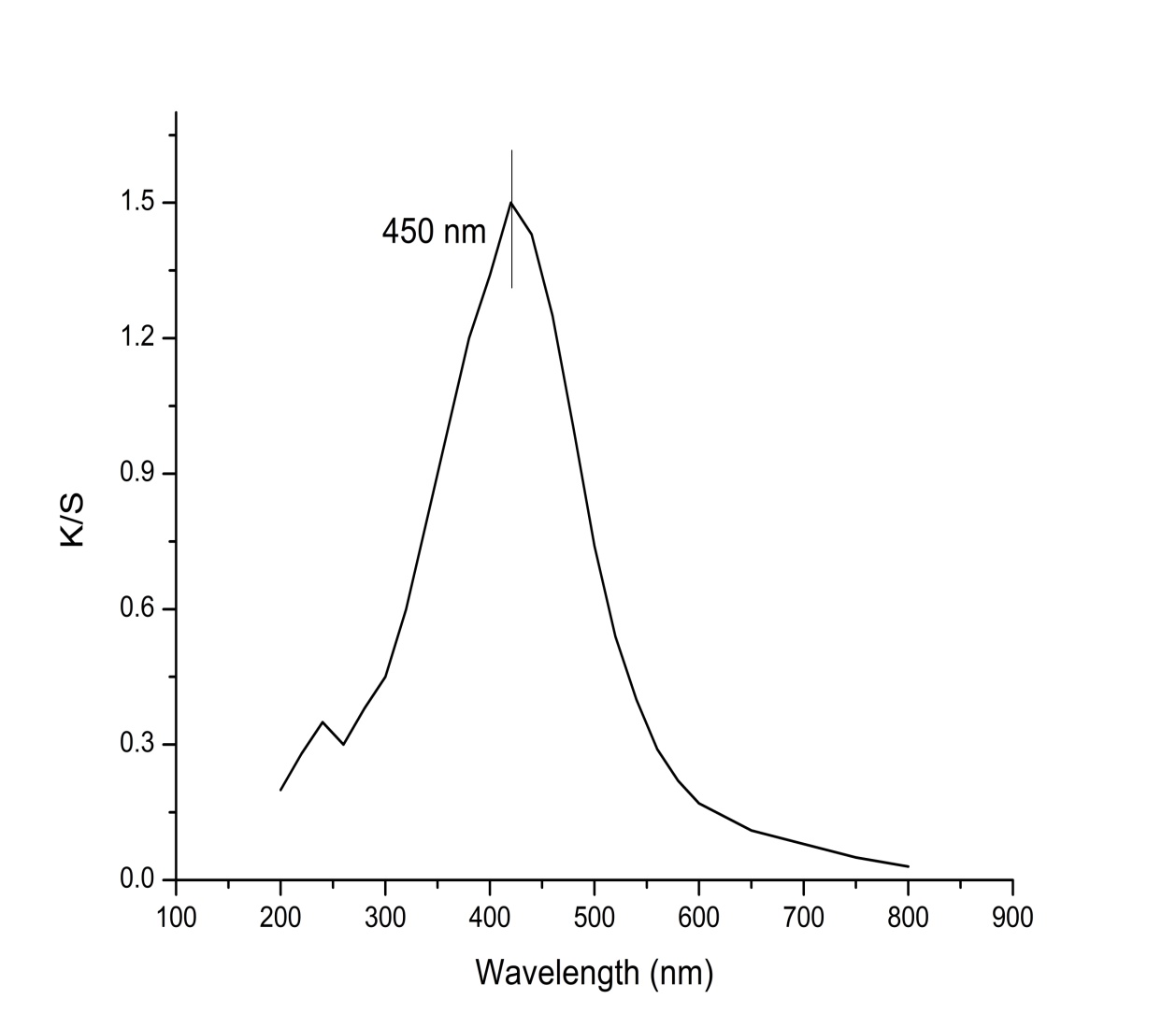


**S4: K/S spectra of the dyed wool and PA6 fabric with natural extracted pigment**

Supplement: Supplementary file 2 — Supplementary Material 4 [file 41598_2026_55888_MOESM2_ESM.docx]

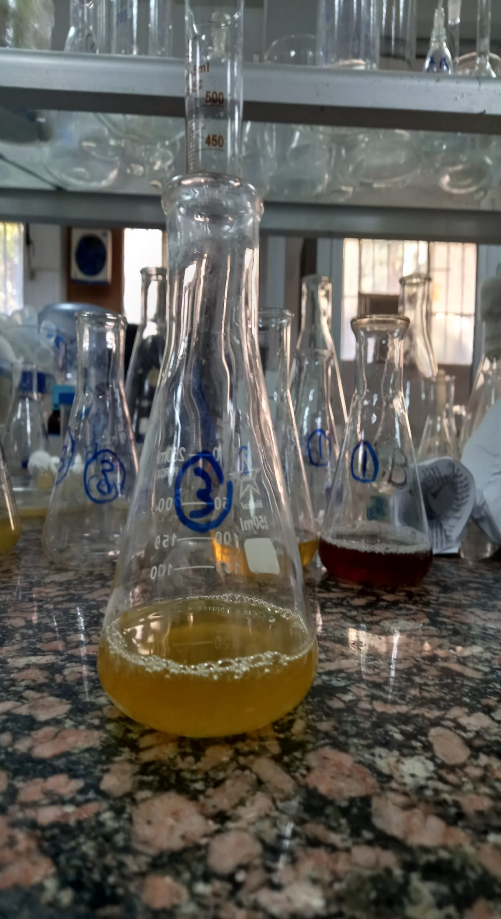

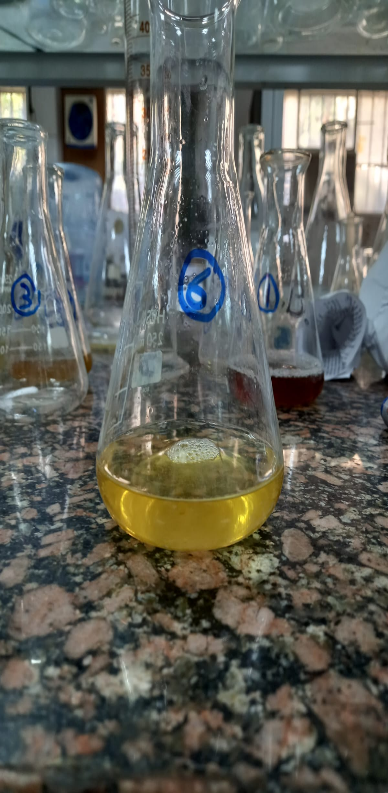

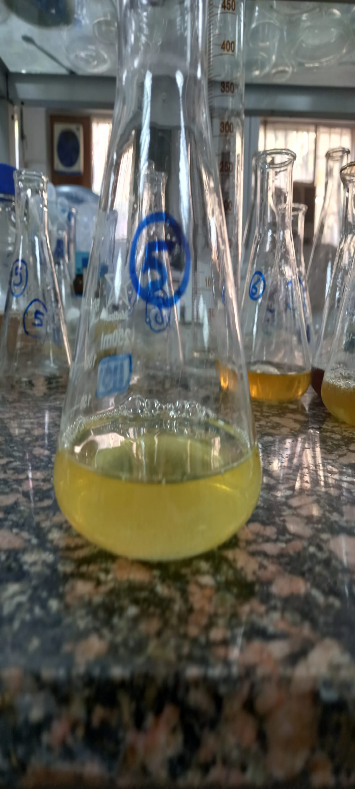

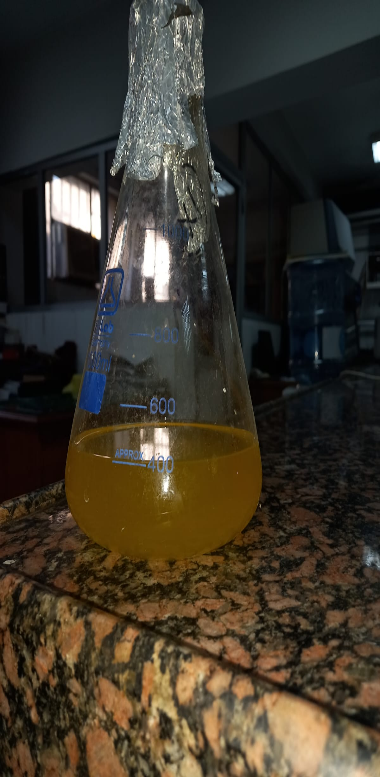


**d)**

**b)**

**c)**

**a)**

**S2:** Effect of incubation temperature at a)25 °C, b) 30 °C, c) 35 °C, and d) 40°C , on pigment production.

Supplement: Supplementary file 4 — Supplementary Material 2 [file 41598_2026_55888_MOESM4_ESM.docx]
