# Supplementary material for: A green approach to antibacterial and antioxidant wool and polyamide 6 fabrics through bioactive Aspergillus turcosus extracted pigment for healthy and high-performance textile products
Source: Sci Rep. 2026 Jun 19;16:19168. doi: 10.1038/s41598-026-55888-w (PMC13282385; doi:10.1038/s41598-026-55888-w)
Supplement: Supplementary file 3 — Supplementary Material 3 [file 41598_2026_55888_MOESM3_ESM.docx]

**4)**

**3)**


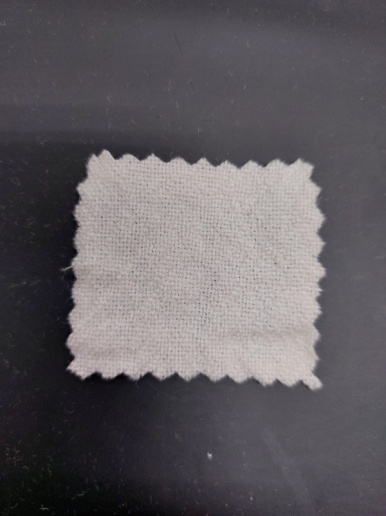

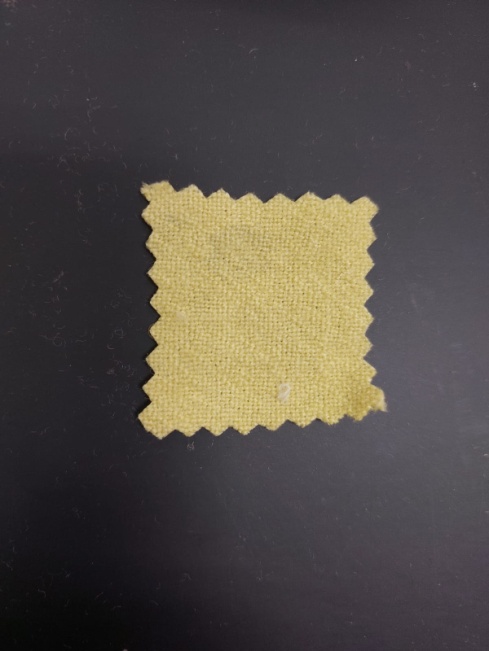

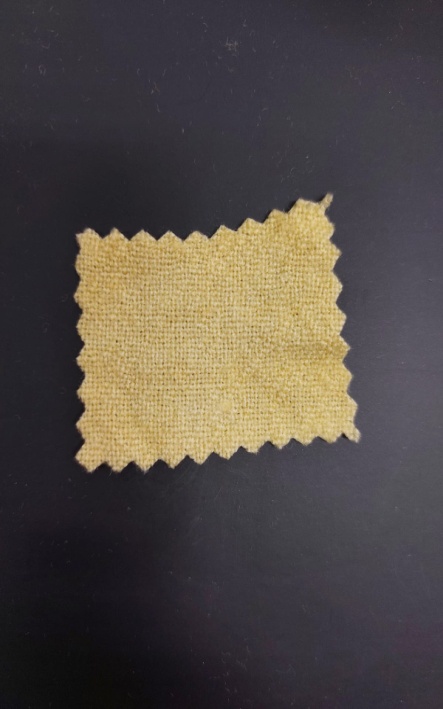

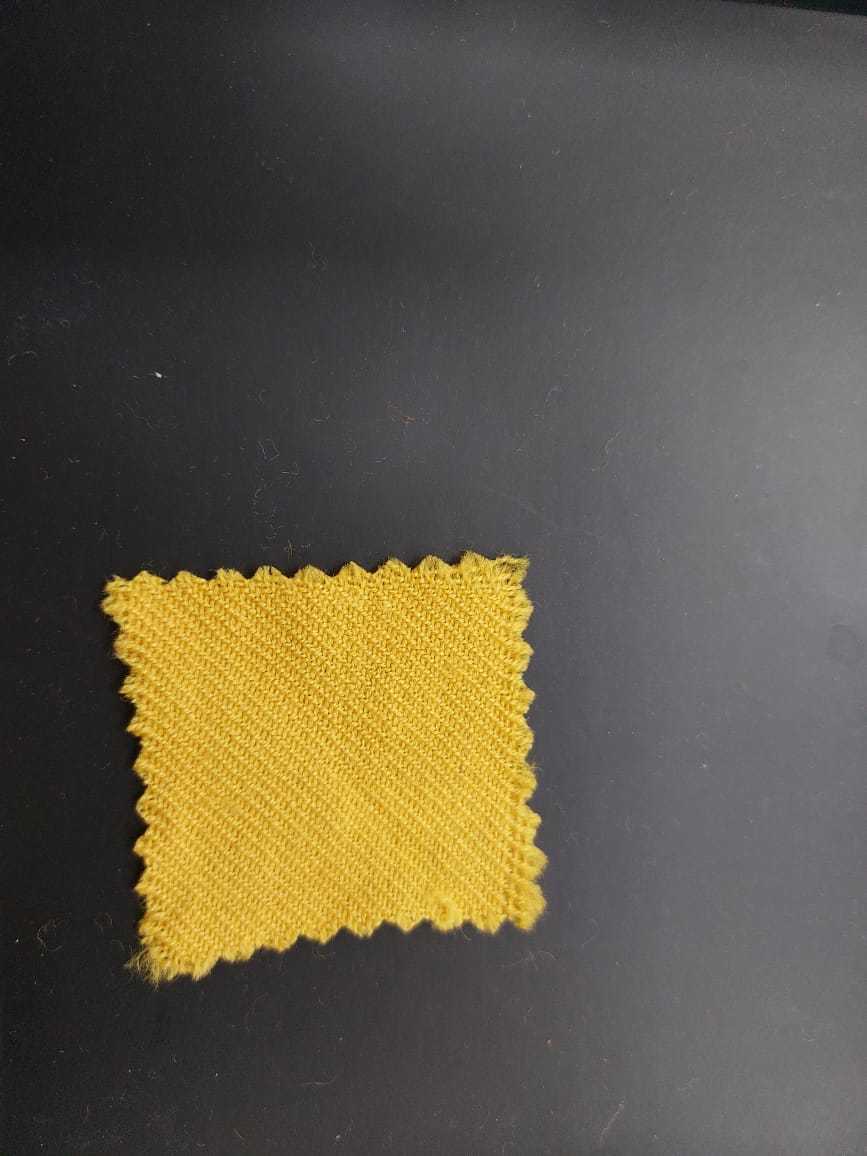


**2)**

**1)**

**5)**

**7)**


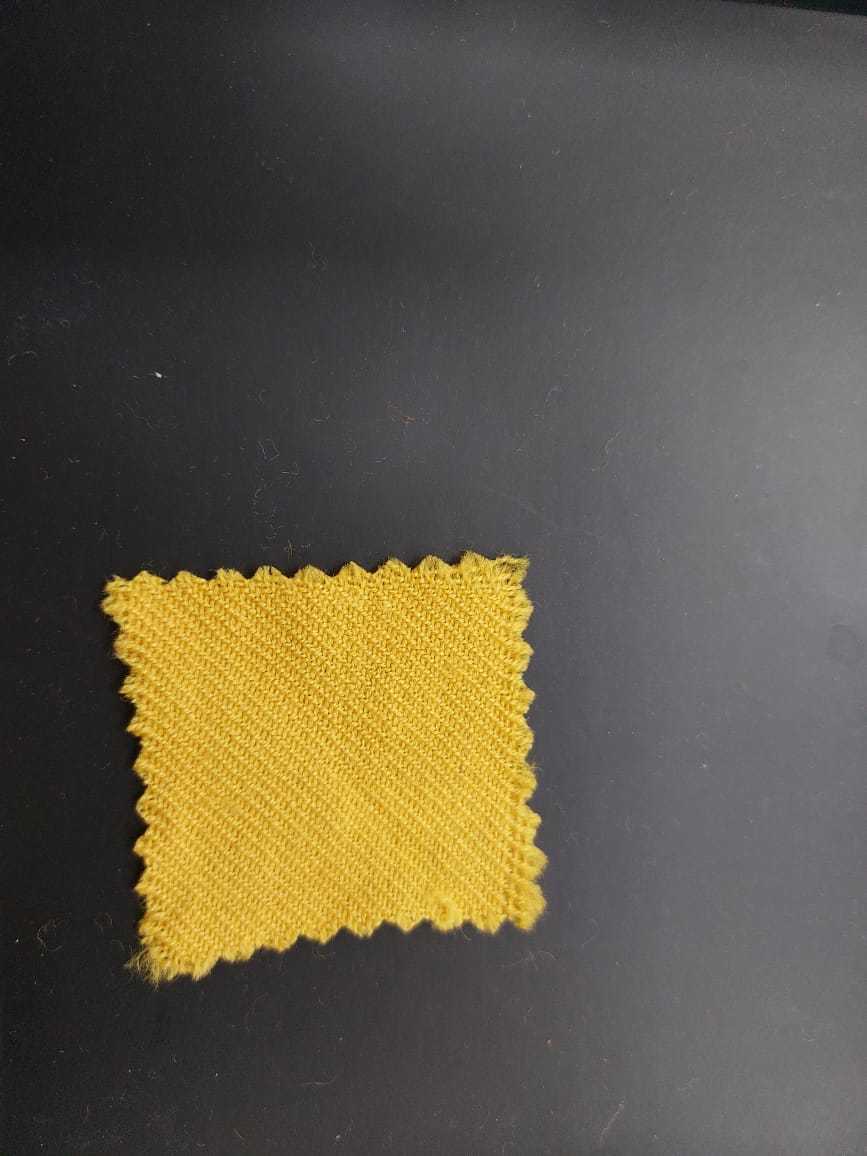

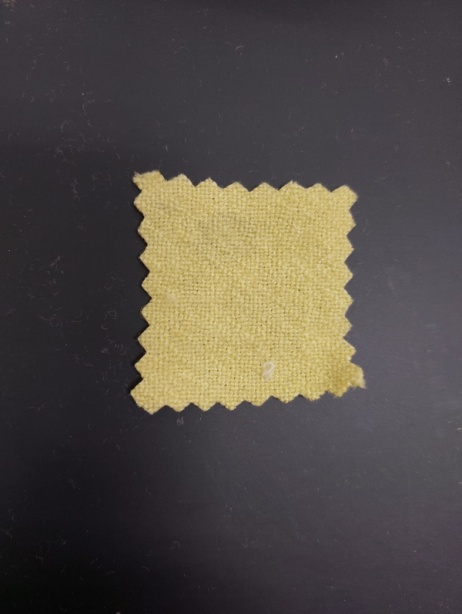

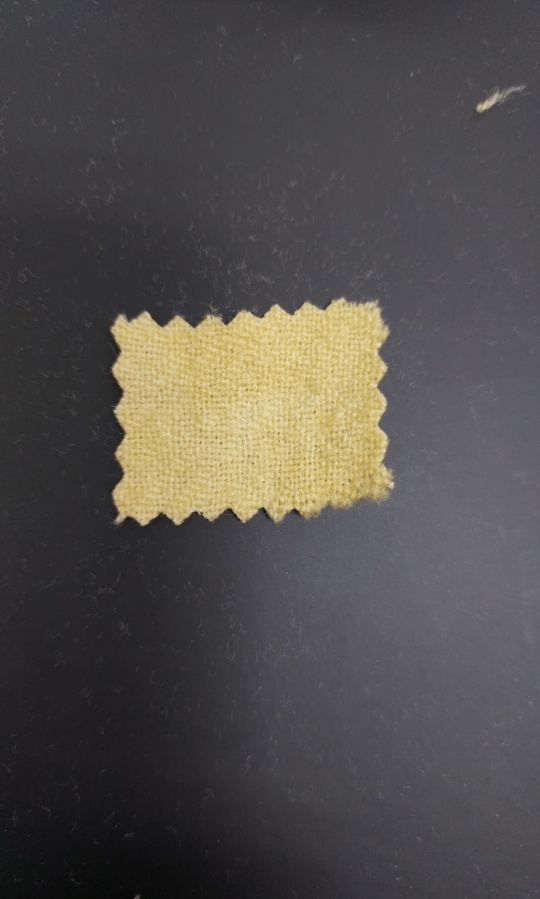


**6)**

**S3-1**: Images of 1) Blank and dyed wool fabrics with 2) 1% shade, 3) 2% shade, 4)3% shade of the extracted pigment in pH 4, at 80 °C for h hr, and dyed wool fabrics at 5)pH 4, 6)pH 7, 7)pH 9

**2)**

**1)**


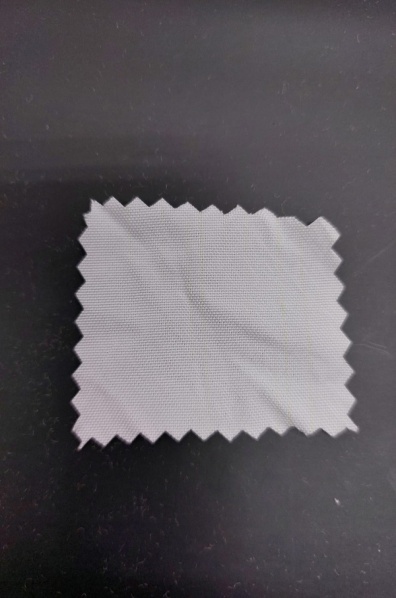

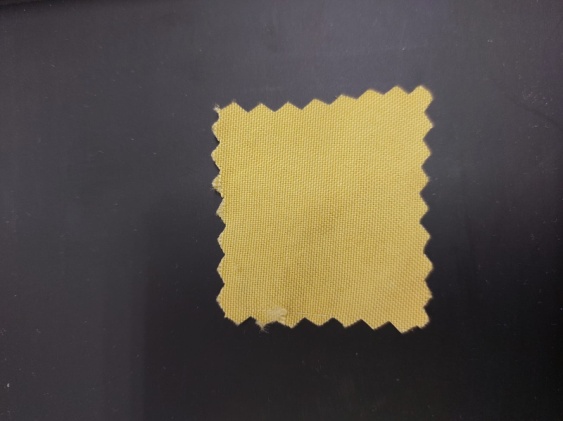

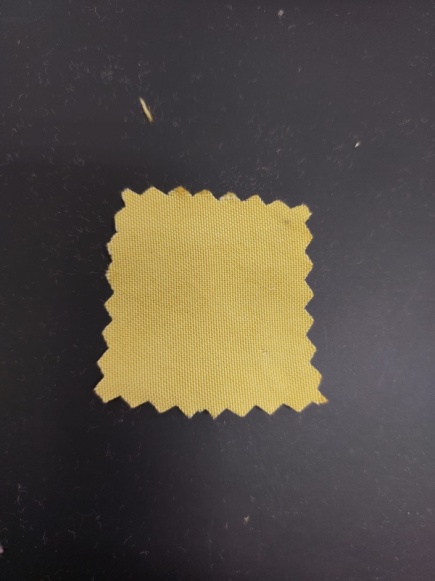

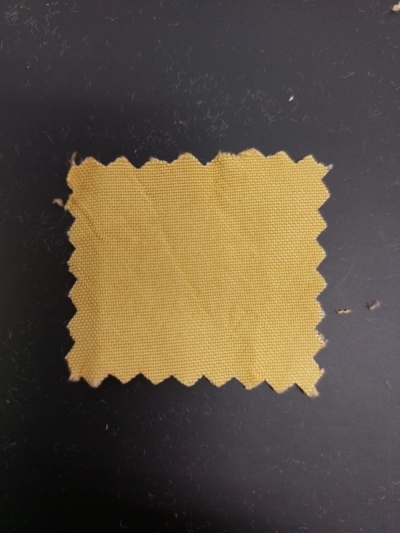


**7)**

**5)**

**4)**

**3)**


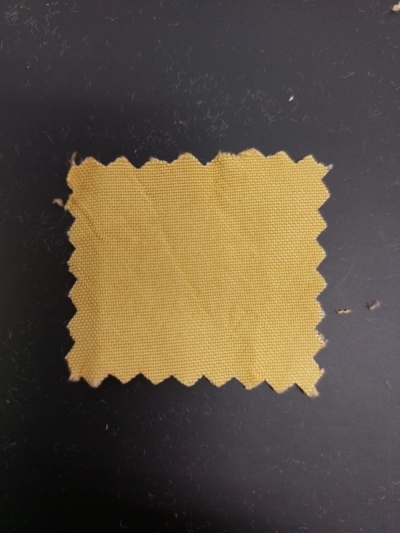

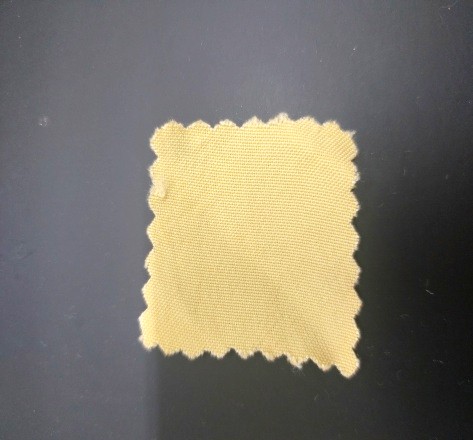

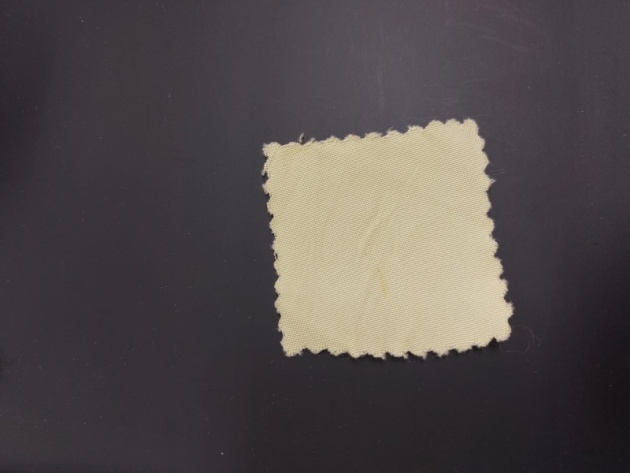


**6)**

**S3-2**: Images of 1) Blank and dyed PA6fabrics with 2) 1% shade, 3) 2% shade, 4)3% shade of the extracted pigment in pH 4, at 80 °C for h hr, and dyed wool fabrics at 5)pH 4, 6)pH 7, 7)pH 9
